# Salivary Proteomics Reveals Disease- and Therapy-Associated Molecular Patterns in Relapsing-Remitting Multiple Sclerosis

**DOI:** 10.3390/biom16101524

**Published:** 2026-10-19

**Authors:** Giulia Guadalupi, Cristina Contini, Chiara Piras, Giada Pisano, Jessica Frau, Giuseppe Fenu, Giancarlo Coghe, Greca Lai, Massimo Castagnola, Irene Messana, Giacomo Diaz, Tiziana Cabras, Alessandra Olianas, Eleonora Cocco, Barbara Manconi, Lorena Lorefice

**Affiliations:** 1 Department of Life and Environmental Sciences, University of Cagliari, 09121 Cagliari, Italy; giulia.guadalupi@unica.it (G.G.); cristina.contini93@unica.it (C.C.); greca.lai@unica.it (G.L.); imessana53@gmail.com (I.M.); tcabras@unica.it (T.C.); olianas@unica.it (A.O.); 2 Multiple Sclerosis Center, Binaghi Hospital, ASL Cagliari, University of Cagliari, 09126 Cagliari, Italy; chiarapiras95@gmail.com (C.P.); giada.pisano.neuro@gmail.com (G.P.); jessicafrauneuro@gmail.com (J.F.); giusefenu@gmail.com (G.F.); gccoghe@gmail.com (G.C.); ecocco@unica.it (E.C.); 3 Fondazione Policlinico Universitario Agostino Gemelli IRCCS, 00168 Rome, Italy; maxcastagnola@outlook.it; 4 Department of Biomedical Sciences, University of Cagliari, 09121 Cagliari, Italy; gdiaz@unica.it

**Keywords:** relapsing-remitting multiple sclerosis, saliva, proteomics, mass spectrometry, disease-modifying therapy, longitudinal monitoring, machine learning

## Abstract

Multiple sclerosis (MS) is a chronic immune-mediated disease for which non-invasive biomarkers, able to detect disease- and treatment-associated molecular changes, are needed. We characterized the whole-saliva proteome of patients with relapsing-remitting (RR) MS at baseline before treatment initiation (MS-T0; n = 66) and after six months of disease-modifying therapy (MS-T1; n = 40), in comparison with healthy controls (HC; n = 31). Nano-liquid chromatography-high-resolution tandem mass spectrometry identified 1305 proteins, of which 209 differed among the main comparisons. Pairwise and paired analyses distinguished persistent RRMS-associated alterations, baseline-attenuation patterns with changes shifting towards HC levels, treatment-associated differences emerging at MS-T1, and longitudinal-only alterations. The identified patterns are descriptive and cannot be interpreted as evidence of pharmacological treatment effects or clinical response. The existence of specific RRMS alterations was supported by exploratory Random Forest, which classified HC, MS-T0 and MS-T1 samples using a panel of 50 proteins, most of which (96%) also showed significant changes in univariate tests. Reactome enrichment evidenced that proteins pertaining to the identified patterns are mainly involved in innate immunity, neutrophil degranulation, cell-death pathways, cytokine signalling, and protein folding. These findings indicate that six months of therapy was associated with substantial salivary proteome changes and support saliva as a candidate biofluid for longitudinal molecular monitoring and non-invasive detection of treatment-associated salivary proteome variations in RRMS.

## 1. Introduction

Multiple sclerosis (MS) is a chronic immune-mediated disease of the central nervous system (CNS) and one of the leading causes of neurological disability in young adults [1]. Its pathological substrate includes inflammatory demyelination, axonal injury, gliosis and neurodegeneration, which may coexist to variable degrees across patients and disease stages [1,2]. This biological heterogeneity is particularly relevant in relapsing-remitting MS (RRMS), where clinical relapses and magnetic resonance imaging (MRI) activity provide essential information for disease monitoring but only partially reveals the molecular processes underlying disease evolution and treatment response [2,3].

The therapeutic landscape of MS has expanded substantially in recent decades, with disease-modifying therapies (DMTs) targeting different components of immune activation, lymphocyte trafficking, B-cell function and inflammatory signalling. Although these treatments have improved disease control and long-term outcomes, inter-individual variability in treatment response remains substantial. Consequently, there is a growing need for molecular biomarkers able to complement clinical and radiological assessment, support patient stratification, and distinguish biological changes occurring during treatment exposure [4,5].

Fluid biomarkers have gained increasing relevance in MS because they can provide dynamic information on disease activity, neuroaxonal damage and treatment response. Cerebrospinal fluid (CSF) remains highly informative for diagnostic and pathophysiological assessment, while blood-based biomarkers, particularly neurofilament light chain, have proven useful in reflecting disease activity, neuroaxonal injury and therapeutic response [4,5]. However, both CSF and blood-based approaches present limitations for repeated longitudinal sampling, due to invasiveness or variability introduced by systemic factors. Therefore, additional non-invasive biofluids could provide complementary information for molecular monitoring in MS.

Proteomics is particularly well-suited for biomarker discovery, because proteins represent functional effectors of biological processes and direct mediators of immune, metabolic and cellular stress responses. Previous proteomic studies on MS, mainly performed on CSF and blood, have identified alterations involving immune regulation, oxidative stress, cytoskeletal remodelling, metabolic pathways and inflammatory signalling [6,7]. These findings support the potential of proteomics not only to identify candidate biomarkers, but also to provide insight into disease-associated biological processes. Nevertheless, longitudinal proteomic studies capable of distinguishing persistent disease-associated alterations from those resulting from treatment-induced molecular remodelling remain limited.

Saliva is an attractive biofluid for longitudinal biomarker studies because it can be collected easily, repeatedly and non-invasively. The salivary proteome includes proteins derived from local oral secretions as well as components related to systemic circulation and inflammatory processes, making saliva a potentially informative matrix for monitoring systemic and immune-mediated diseases [8]. Salivary biomarkers have been explored in neurological and immune-mediated disorders [9,10,11,12], and previous work has shown that salivary proteomic profiling can detect disease-associated molecular differences in MS [13]. However, the ability of salivary proteomics to identify longitudinal changes in RRMS patients after DMT exposure remains underexplored.

The integration of mass spectrometry-based proteomics with multivariate machine-learning approaches may improve the identification of biologically meaningful protein signatures. While univariate statistics identify proteins showing significant abundance differences between groups, machine-learning models can identify coordinated multivariate patterns that contribute to sample classification. In this context, Random Forest classification combined with Boruta feature selection provides a useful framework to identify proteins with discriminative values and to relate classification performance to biologically coherent molecular modules.

In the present study, we investigated the whole saliva proteomic profile of RRMS patients at baseline before treatment initiation and after six months of DMT, in comparison with HC. By integrating nanoLC– high-resolution MS/MS, differential abundance analysis, Random Forest classification with Boruta feature selection, proteomic patterns analysis and Reactome pathway enrichment, we aimed to identify persistent RRMS-associated salivary alterations, proteins displaying abundance shifts towards HC levels after treatment exposure, and treatment-associated proteomic remodelling. This approach was designed to explore the potential of saliva as a non-invasive biofluid for longitudinal molecular monitoring in RRMS.

## 2. Materials and Methods

### 2.1. Participants

Sixty-six patients with RRMS, diagnosed according to the 2017 revised McDonald criteria [14] were enrolled at the Multiple sclerosis Center of Cagliari between 2022 and 2023. The MS-T0 cohort included 66 patients who were either treatment-naïve or “switchers,” the latter defined as patients transitioning from first-line therapies (interferons, glatiramer acetate, dimethyl fumarate, or teriflunomide) because of suboptimal efficacy, with these treatments discontinued at least one month before enrollment. This interval reflected clinical practice and the need to avoid prolonged periods without therapeutic coverage. Patients switching from teriflunomide to second-line therapies underwent an accelerated elimination procedure, as described by the European Medicines Agency (EMA). Immediately after the first sample collection, patients were assigned to pharmacological treatment according to Italian Medicines Agency recommendations. Patients with active disease, defined as at least one clinical relapse within the previous year and/or evidence of MRI activity on the most recent scan, including gadolinium-enhancing lesions or new or enlarging T2 lesions, initiated a high-efficacy DMT, whereas patients with moderate disease activity received first-line treatments. After six months of treatment, 40 of the 66 patients returned for follow-up (MS-T1), and a second saliva sample was collected. Of these, 16 received high-efficacy DMTs (cladribine, n = 5; ocrelizumab, n = 6; ozanimod, n = 2; natalizumab, n = 3), while 24 received first-line treatments (teriflunomide, n = 11; dimethyl fumarate, n = 13). Thirty-one healthy controls (HCs), recruited from the local population, were matched to the overall MS-T0 cohort by age and sex. Because follow-up samples were available for a subset of patients, demographic comparability was additionally assessed for the MS-T1 subgroup.

All participants, both patients and controls, provided written informed consent in accordance with the latest version of the Declaration of Helsinki. The study was approved by the local Ethics Committee (Prot. 417-2022/CE).

Clinical data for RRMS patients, including disease duration and disability level, assessed using the Expanded Disability Status Scale (EDSS) [15], were collected prior to the initiation of DMT.

Exclusion criteria for RRMS patients included the presence of chronic comorbidities (including major oral diseases), corticosteroid treatment within the previous 30 days, or chronic medication use. Similarly, HC were required to have no chronic or autoimmune diseases, major oral conditions, chronic medication use, or family history of RRMS.

### 2.2. Sample Collection

Unstimulated whole saliva (WS) was collected the morning after participants had fasted from both food and beverages, including coffee, for at least 3 h. Participants were required to have brushed their teeth at least 10 min before collection and not to wear lipstick or lip balm or to have used chewing gum, candies or similar products.

Participants remained seated in a relaxed position and allowed saliva to accumulate under the tongue and between the cheeks and mandible. Approximately 0.5 mL of WS was collected using a plastic Pasteur pipette. During collection, the tube was maintained on ice. Any oral inflammatory condition or minor oral lesion observed or reported at the time of collection, and smoking status were recorded and documented.

Immediately after collection, all samples were diluted 1:1 (*v*/*v*) with phosphate-buffered saline (PBS: 270 mM NaCl, 5 mM KCl, 20 mM Na_2_HPO_4_, 4 mM KH_2_PO_4_) supplemented with 25× cOmplete™ ULTRA Tablets, Mini, EDTA-free EASYpack Protease Inhibitor (Sigma-Aldrich/Merck, Darmstadt, Germany). Samples were then stored at −80 °C until further analysis.

Prior to protein quantification, samples were gently centrifuged at 1000× *g* for 5 min at 4 °C. Total protein concentration was measured in duplicate for each sample using the Pierce™ BCA Protein Assay Kit (Thermo Fisher Scientific, Waltham, MA, USA), following the manufacturer’s instructions.

### 2.3. Enzymatic Digestion

A total of 25 μg of total protein from each WS sample was subjected to an in-solution enzymatic digestion protocol. Samples were initially diluted in 0.2% RapiGest™ SF surfactant (Waters™, Milford, MA, USA) and 10 mM dithiothreitol (DTT), then boiled at 100 °C for 5 min, followed by incubation at 65 °C for 30 min to reduce disulfide bonds.

Alkylation of cysteine residues was carried out by incubating the samples with 55 mM iodoacetamide at room temperature in the dark. The reaction was subsequently quenched by adding DTT to a final concentration of 50 mM.

Proteolytic digestion was performed by adding Pierce™ Trypsin Protease, MS Grade (Thermo Fisher Scientific, Waltham, MA, USA) at an enzyme-to-substrate ratio of 1:50 (*w*/*w*). Samples were incubated overnight at 37 °C. The enzymatic reaction was terminated by the addition of formic acid (FA) to a final concentration of 0.1%.

Prior to mass spectrometry analysis, tryptic peptides were desalted using OMIX C18 100 μL pipette tips (Agilent Technologies, Santa Clara, CA, USA) according to the manufacturer’s instructions. The desalted peptide mixtures were then freeze-dried and reconstituted in 0.1% FA for mass spectrometry analysis.

### 2.4. Nano-LC–High-Resolution ESI-MS/MS Analysis

All reagents and chemicals were purchased from Sigma-Aldrich (St. Louis, MO, USA), unless otherwise specified. A measure of 1 μg of total peptides from each sample was analyzed in a randomized sample injection order to minimize analytical bias using an Ultimate 3000 Nano LC system (Thermo Fisher Scientific Waltham, MA, USA) coupled to a high-resolution LTQ Orbitrap Elite mass spectrometer (Thermo Fisher Scientific Waltham, MA, USA). Peptide separation was carried out on an EASY-Spray™ reverse-phase C18 nanoLC column (250 mm × 75 μm inner diameter, 2 μm particle size; Thermo Fisher Scientific). Peptides were eluted over 100 min at a flow rate of 0.3 μL/min using a gradient consisting of solvent A (0.1% FA in water) and solvent B (0.1% FA in acetonitrile (ACN)/water 80/20 *v*/*v*), under the following conditions: 0–3 min: 4% B; 3–80 min: 4–50% B; 80–90 min: 50–80% B; 90–92 min: 80–90% B; 92–100 min: hold at 90% B.

The mass spectrometer was operated in data-dependent acquisition mode with electrospray ionization (ESI) at 1.5 kV. The capillary temperature was set to 275 °C, and the S-Lens RF level to 67.99%. Full MS scans were acquired in positive ion mode over a mass range of *m*/*z* 350–1600 with a resolution of 120,000 (at *m*/*z* 400).

The 10 most intense precursor ions from each full scan were selected for collision-induced dissociation fragmentation with the following parameters: normalized collision energy of 35%, activation time of 10 ms, isolation width of 2 *m*/*z*, and activation q of 0.25.

Raw data were visualized and managed using Xcalibur software version 2.2 SP1.48 (Thermo Fisher Scientific).

Protein identification was performed using Proteome Discoverer software (version 2.2; Thermo Fisher Scientific) with the integrated SEQUEST HT search engine (University of Washington, licensed to Thermo Electron Corporation, San Jose, CA, USA). Spectra were searched against the reviewed *Homo sapiens* entries in the UniProtKB/Swiss-Prot database (20,404 entries, release 2022_05).

Search parameters were set as follows: Fixed modification: carbamidomethylation of cysteine; Dynamic modifications: methionine oxidation, serine/threonine phosphorylation, N-terminal pyroglutamate formation, and N-terminal acetylation; Enzyme specificity: trypsin, allowing up to two missed cleavages; Peptide mass tolerance: 10 ppm; Fragment ion mass tolerance: 0.6 Da.

Peptides were filtered for high confidence, with a minimum sequence length of 6 amino acids. False discovery rate (FDR) thresholds were set at 0.01 (strict) and 0.05 (relaxed).

Only proteins identified with at least two unique peptides and passing high-confidence FDR thresholds were retained for further analysis. Common contaminants, including keratins, were excluded.

Protein quantification was performed using the label-free quantification module within Proteome Discoverer, based on the precursor ion area of unique peptides. Protein abundances were normalized with respect to the total amount of tryptic peptides using the Precursor Ions Quantifier node.

All mass spectrometry proteomics data have been deposited in the ProteomeXchange Consortium via the PRIDE partner repository [16] under the dataset identifier PXD053357.

### 2.5. Statistical Analysis

Only proteins with abundances above the limit of detection (LOD) in at least 30% of samples within each group were retained for statistical analysis. Prior to analysis, data were log10-transformed, then values below the LOD were imputed using the imputeQRILC method, based on quantile regression, available in the imputeLCMD R package [17]. Data were quantile-normalized and processed for group comparisons by non-parametric tests, given considerable deviation from normality.

Because only a subset of patients returned for follow-up sampling after six months of treatment (n = 40), both paired and unpaired comparisons were performed. The paired analysis evaluated longitudinal changes within the same individuals, whereas the unpaired analysis compared the entire baseline cohort (n = 66) with the follow-up cohort (n = 40). Specifically, we used (a) the Wilcoxon rank-sum test for independent samples to compare groups of different individuals (MS-T0, MS-T1 and HC) and (b) the Wilcoxon signed-rank test for paired samples to compare measurements within the same individual at baseline and at the 6-month follow-up. Multiple *p*-values were verified by Benjamini–Hochberg procedure, to maintain an FDR below 1% [18]. Venn diagrams were generated using the jvenn tool [19] to illustrate the overlap of upregulated and downregulated proteins across groups. All 137 samples were processed by Random Forest (RF), independently from the fact that 40 MS-T0 and MS-T1 samples were from the same patients. The Boruta algorithm [20] was used to perform a preliminary selection of proteins with discriminating power between the different groups, to submit to RF. Boruta was selected because the exploratory objective was to identify an all-relevant set of proteins contributing to group discrimination, including correlated features and nonlinear relationships. The RF model comprised 1500 decision trees and was validated using the out-of-bag (OOB) error estimate, that is the error in classification using, for each decisional tree, two-thirds of data as the training set, and the remaining one-third as the validation set. The importance of each protein in the classification of samples was assessed by the ‘mean decrease accuracy’, that is the decrease in the classification performance after the exclusion of that protein from the procedure. Classification results were represented by confusion matrices and multidimensional scaling (MDS) plots. All statistical analyses were performed in R [21]. Settings and parameters for the statistical analyses and RF are reported in the Supplementary File S1.

Correlation between salivary protein abundances and demographic and clinical variables (year of disease onset, age at disease onset, age at baseline, disease duration at baseline, EDSS score) was assessed using Spearman’s rank correlation. Correlations were calculated separately for protein abundances at MS-T0 and MS-T1. *p*-values were adjusted for multiple testing within each analysis using the Benjamini–Hochberg procedure, and correlations with a false discovery rate below 5% were considered statistically significant.

To evaluate whether previous DMT exposure could have influenced the baseline salivary proteome, an exploratory sensitivity analysis was performed at MS-T0 by comparing treatment-naïve patients (n = 18) and previously treated switchers (n = 48). Protein abundances were compared using the Wilcoxon rank-sum test, with Benjamini–Hochberg correction across the 623 proteins.

Heatmaps based on row-wise, standardized abundances were generated using all proteins showing significant variation across the main comparisons and on the set of proteins selected by Boruta algorithm for RF.

### 2.6. Pathway Enrichment Analysis

Reactome pathway enrichment analyses were performed using g:Profiler g:GOSt (version e111_eg58_p18_30541362; database last updated on 25 January 2024). UniProt accession codes were used as input identifiers, *Homo sapiens* was selected as the target organism, and Reactome was selected as the only functional annotation source. Pathways with FDR-adjusted *p*-values < 0.05 were considered significantly enriched.

To evaluate the directional contribution to each enriched pathway, UniProt accession codes reported in the g:Profiler intersection output were manually cross-referenced with the direction of change reported in Supplementary Tables S2 and S4. Reactome enrichment analysis was also performed on the proteins selected by the Boruta algorithm to assess the biological coherence of this discriminant protein set with respect to the entire set of statistically varied proteins.

## 3. Results

### 3.1. Characteristics of the Participants

Table 1 summarizes the demographic and clinical features of MS-T0 and MS-T1 patients and HC. No significant differences in age or sex distribution were observed between HC and the overall MS-T0 and MS-T1 cohorts.

Complete demographic and clinic data of patients and controls are reported in Supplemental Table S1a,b. A comparison of baseline characteristics between participants who completed the six-month follow-up and those who did not is provided in Supplementary Table S1c.

An exploratory sensitivity analysis compared baseline salivary protein abundances between treatment-naïve patients (n = 18) and switchers (n = 48). Of the 623 proteins tested, 54 showed nominal *p*-values < 0.05, but none remained significant after Benjamini–Hochberg correction (minimum adjusted *p* = 0.456; Supplementary Table S1d).

### 3.2. Global Salivary Proteomic Alterations in RRMS Patients

Shotgun proteomic analysis enabled the high-confidence identification of a total of 1305 proteins after the removal of contaminants; of these, 623 met the criteria for statistical analysis. Using the Wilcoxon rank-sum test, 209 proteins showed significantly different abundances in the comparison between the three groups, with test *p*-values < 0.05 and an FDR < 1% (Supplementary Table S2).

**Figure 1:**
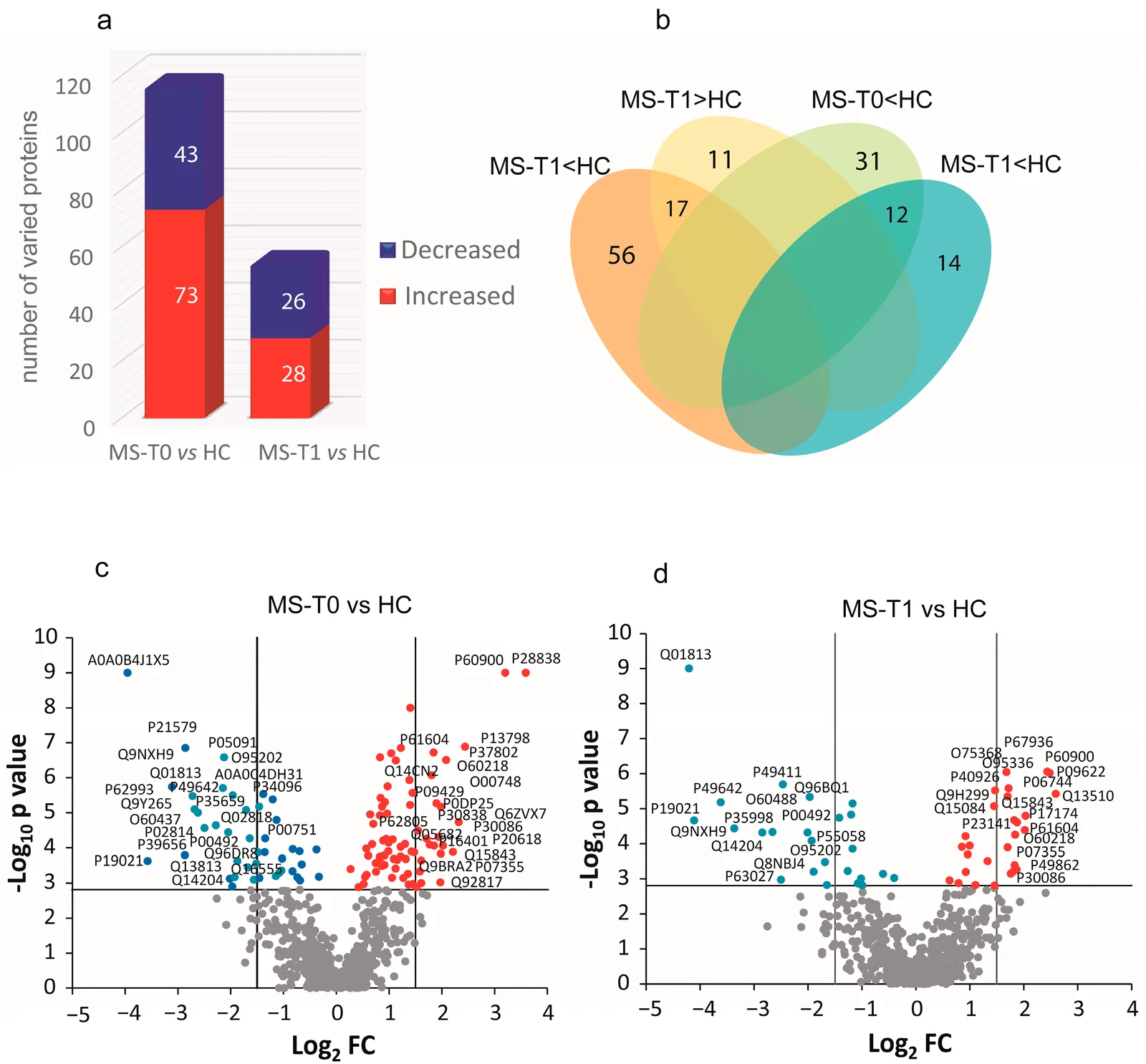
Global salivary proteomic alterations in RRMS patients. (**a**) Bar chart showing the number of proteins differentially abundant in MS-T0 and MS-T1 RRMS patients compared to HC. Red bars indicate proteins with increased abundance and blue bars indicate proteins with decreased abundance in patients. (**b**) Venn diagram showing the overlap among proteins significantly altered in the four comparisons (MS-T0 > HC, MS-T0 < HC, MS-T1 > HC, and MS-T1 < HC). (**c**) Volcano plot of the comparison between MS-T0 and HC. (**d**) Volcano plot of the comparison between MS-T1 and HC. Significantly increased proteins are shown in red, significantly decreased proteins in blue, all others in grey. The horizontal line at -Log10 *p*-value = 2.8 corresponds to the threshold of significant *p*-values (*p* < 0.001552, with a FDR < 1%). The vertical lines correspond to the Log2 FC threshold of <−1.5 or >1.5. Only the proteins falling within these *p*-value and FC ranges are labelled.

Specifically, 116 proteins were differentially abundant in MS-T0 patients compared with HC, with a 63% increase in the patients, whereas only 54 proteins differed between MS-T1 patients and HC, with a 52% increase in the patients (Figure 1a). The number of shared and unique proteins across the comparisons (MS-T0 > HC, MS-T0 < HC, MS-T1 > HC, and MS-T1 < HC) is shown in the Venn diagram (Figure 1b), while the complete lists of the corresponding protein subsets are reported in Supplementary Table S2. Only 17 proteins with increased levels and 12 with decreased levels were common to the MS-T0 and MS-T1 groups compared to HC.

The results of MS-T0 vs. HC and MS-T1 vs. HC comparisons are summarized in the volcano plots shown in Figure 1c,d.

Among the proteins showing the largest fold change in the MS-T0 compared to HC, Cytosol aminopeptidase (P28838) and Proteasome subunit alpha type-6 (P60900) were increased, whereas Immunoglobulin heavy variable 3-74 (A0A0B4J1X5) was decreased. In the comparison between MS-T1 and HC, ATP-dependent 6-phosphofructokinase (Q01813) was the protein showing the greatest decrease in level.

Results of the Spearman correlation analyses, performed between salivary protein abundance and clinical variables (age at baseline, age at disease onset, year of disease onset, disease duration at baseline and baseline EDSS) are reported in Supplementary Table S2b,c. After Benjamini–Hochberg correction, five proteins showed moderate inverse correlations with age at baseline in MS-T0 samples (Cysteine-rich secretory protein 3 (P54108), Progranulin (P28799), Protein LEG1 homolog (Q6P5S2), SPARC-like protein 1 (Q14515), and Mucin-7 (Q8TAX7)). Eight proteins were significantly associated with age at disease onset: six showed positive correlations (Cysteine-rich secretory protein 3 (P54108), Progranulin (P28799), Protein LEG1 homolog (Q6P5S2), SPARC-like protein 1 (Q14515), and Mucin-7 (Q8TAX7)) and two showed inverse correlations (Protein LEG1 homolog (Q6P5S2) and Cysteine-rich secretory protein 3 (P54108)). No associations with year of disease onset, disease duration at baseline or baseline EDSS remained significant after correction for multiple testing. Similarly, no correlations involving protein abundance at MS-T1 or paired abundance changes between MS-T0 and MS-T1 survived FDR correction.

### 3.3. Machine-Learning Classification of HC, MS-T0 and MS-T1

**Figure 2:**
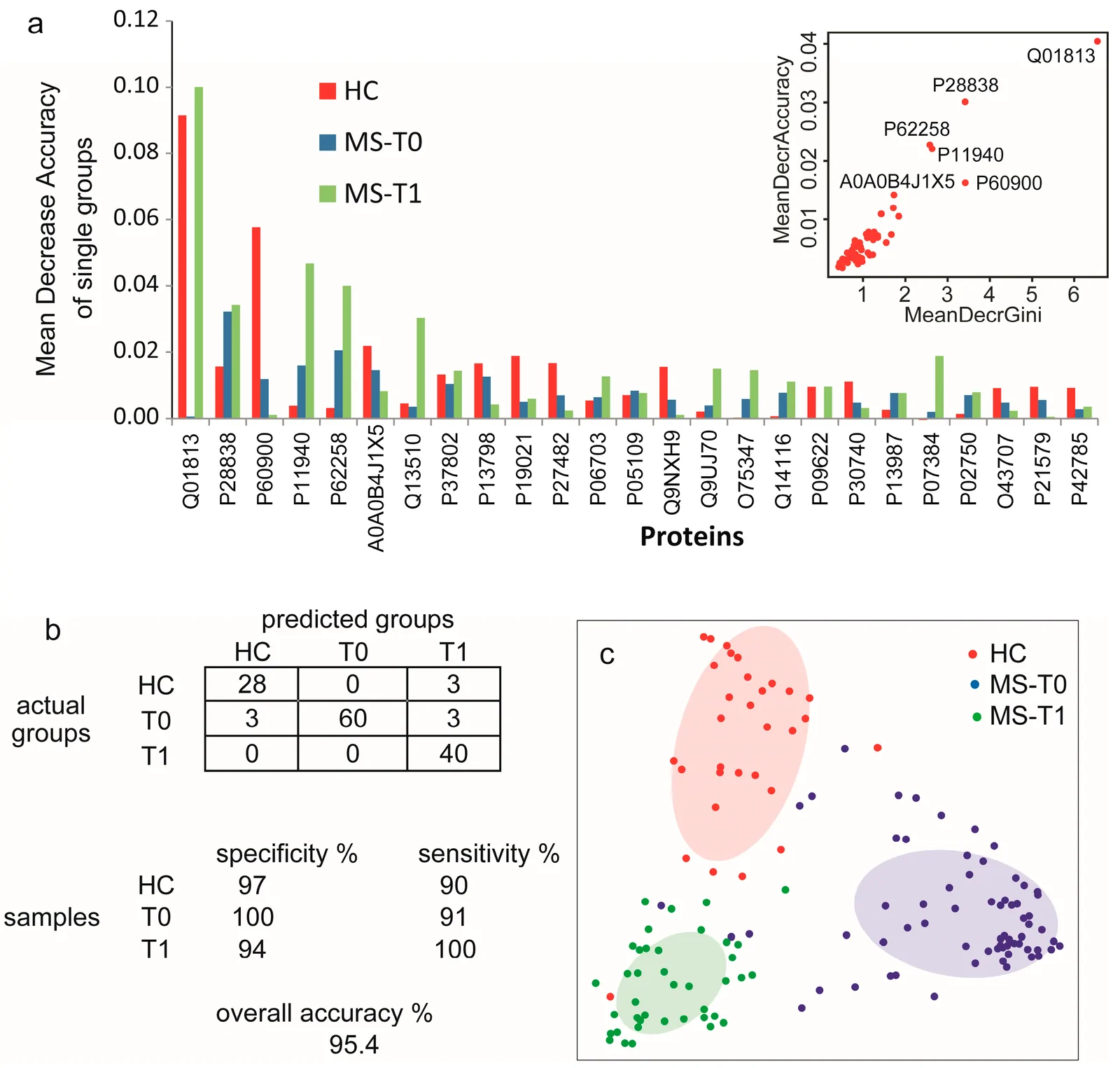
Machine-learning classification of HC, MS-T0 and MS-T1 subjects. (**a**) Mean Decrease Accuracy of the top 25 proteins showing their contribution to the distinct classification of the three groups; in the panel the importance of the 50 proteins selected for Random Forest (RF) classification evaluated by the Mean Decrease Gini and Mean Decrease Accuracy scores. (**b**) Confusion matrix of RF classification. (**c**) Multidimensional scaling (MDS) plot generated from sample proximity data showing the relationships among the three groups. Proximity is a parameter that evaluates the degree of similarity between two samples, determined by monitoring the frequency with which they fall into the same terminal node across all decision trees. Ellipses represent the dispersion around the group centroids with 1.4 standard deviations. Red dots represent HC subjects, blue dots T0-MS patients, and green dots T1-MS patients.

RF classification of MS-T0, MS-T1 and HC was performed using a subset of 50 proteins selected by the Boruta method and listed in Supplementary Table S3. Figure 2a shows the relative importance of the selected proteins for the classification of the three groups of samples, evaluated by the mean decreased Gini purity score (*x*-axis) and the mean decreased accuracy (*y*-axis). These parameters measure the extent to which classification purity and accuracy decrease when the specific protein is excluded from the procedure. The higher the parameter value, the greater the importance of the protein in the model. The panel shows the specific contribution of the proteins to the classification of samples from each group, based on the mean decrease in accuracy. ATP-dependent 6-phosphofructokinase (Q01813), the level of which significantly decreased in both MS-T0 and MS-T1 compared to HC, emerged as the most important protein in the classification of HC and MS-T1 samples. Four other discriminating proteins were: Cytosol aminopeptidase (P28838), showing increased levels in MS-T0 compared to both MS-T1 and HC and found relevant for the classification of MS-T0 and MS-T1 samples; Proteasome subunit alpha type-6 (P60900), important for the classification of MS-T1 samples; Polyadenylate-binding protein 1 (P11940) was relevant for the classifications of MS-T1 samples and 14-3-3 protein epsilon (P62258) relevant for the classifications of HC samples. Almost all proteins selected by RF showed significant level changes in some of the comparisons among the three groups (Supplementary Tables S2 and S3). Only two out of the 50 proteins selected for RF did not result in being significantly changed in univariate analyses. They were Prolylcarboxypeptidase (P42785) and Wiskott–Aldrich syndrome protein family member 2 (Q9Y6W5), in the 31st and 50th place, respectively, in the accuracy rank list. An illustrative classification tree based on the highest-ranked RF proteins is shown in Supplementary Figure S1. The presence of individual proteins at different split points illustrates their conditional and nonlinear contribution to the multivariate classification and helps explain why RF did not always correspond to univariate statistical tests.

As shown by the confusion matrix (Figure 2b), the RF classification was characterized by high specificity and sensitivity (overall accuracy of 95.4%). No errors were found in the classification of MS-T1 subjects (100% true positive), whereas 6 misclassifications were found among MS-T0 subjects (91% true positive), with 3 being attributed to the HC group and 3 to the MS-T1 group, and 3 misclassifications were found among HC subjects (90% true positive), with 3 being attributed to the MS-T1 group. The RF classification can be visualized by the MDS plot (Figure 2c), which represents the first two dimensions accounting for nearly 80% of the whole multidimensional structure (data based on eigenvalues). A more comprehensive visualization, including the third axis and covering 90% of the interrelationships among samples, is available in the 3D MDS animation provided in Supplemental Materials (Video S1). Reactome enrichment analysis, performed on the 50 proteins selected by Boruta (Supplementary Table S3), showed a significant enrichment in immune-related pathways. The most significant term was Neutrophil degranulation, followed by Innate Immune System and Immune System contributing to the classification of HC, MS-T0 and MS-T1 groups and supporting the biological coherence of the machine-learning-derived approach.

### 3.4. Proteomic Patterns Across HC, MS-T0 and MS-T1

Supplementary Figure S2 shows the heatmap generated using the 209 proteins with significant variation among the main pairwise comparisons. Hierarchical clustering suggested three main distinct abundance patterns across HC, MS-T0 and MS-T1 groups that were further defined using the results of statistically significant pairwise comparisons. It is worth noting that the proposed proteins patterns are descriptive and refer to abundance profiles observed across groups and time points in relation to DMT exposure and do not establish a pharmacological effect of DMT, a treatment-specific mechanism or a clinical treatment response.

**Figure 3:**
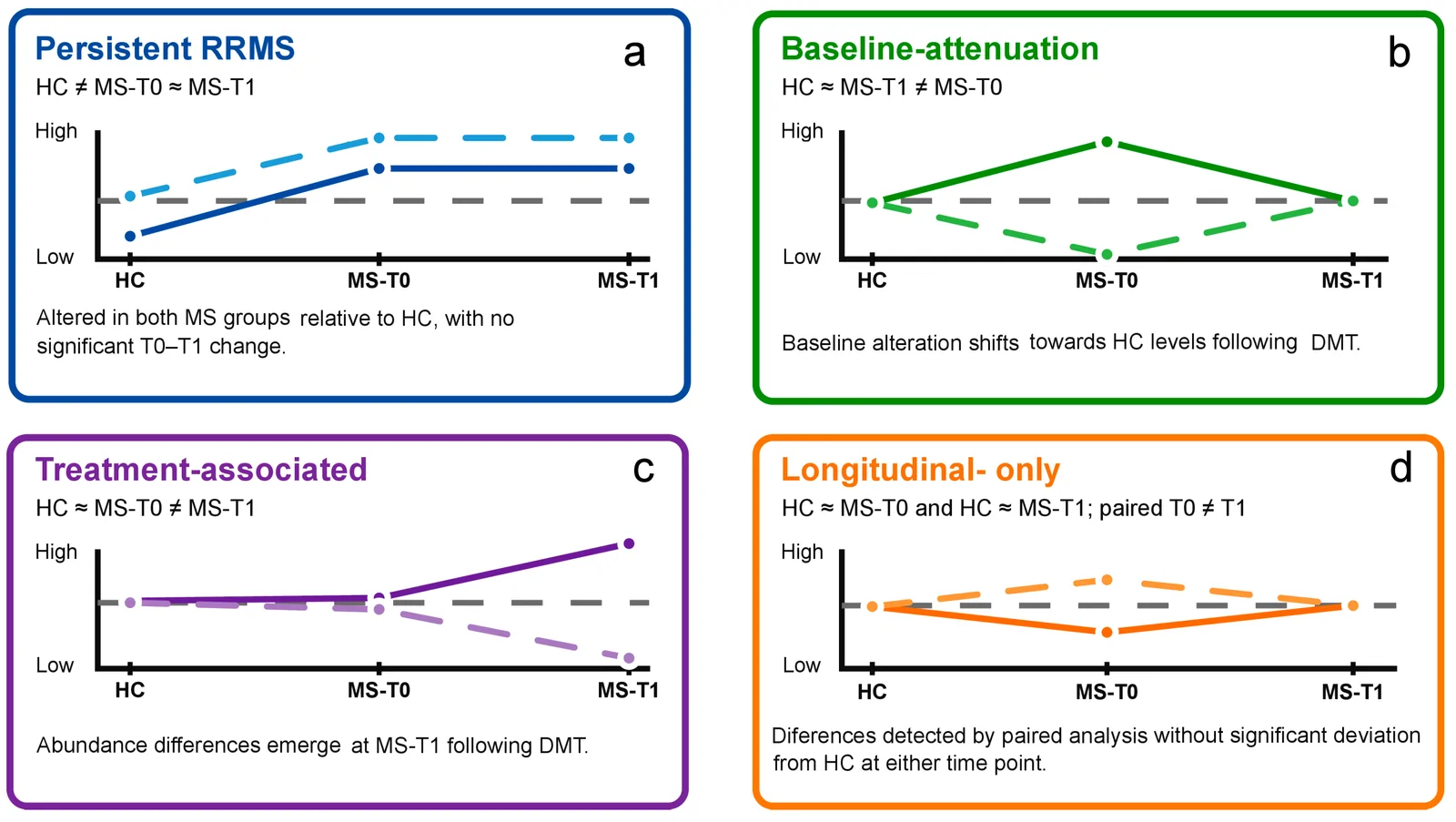
Schematic summary of salivary proteomic patterns in RRMS. Schematic representation of the main abundance patterns identified among HC, MS-T0 and MS-T1. The figure summarizes (**a**) persistent RRMS-associated alterations, (**b**) baseline-attenuation proteins shifting towards HC levels after DMT, (**c**) treatment-associated proteins emerging at MS-T1, and (**d**) longitudinal-only remodelling detected by paired analysis. Solid and dashed lines indicate representative and inverse abundance directions within each pattern. The ‘≈’ symbol stands for ‘no statistically significant differences’, whereas the ‘≠’ symbol stands for ‘statistically significant differences’. Abbreviations: DMT, disease-modifying therapy; HC, healthy controls; RRMS, relapsing-remitting multiple sclerosis.

Twenty-nine proteins (17 proteins with increased levels and 12 with decreased levels) varied in the same direction in both MS-T0 and MS-T1 compared to HC. Of these, 28 proteins (16 increased and 12 decreased) did not vary significantly between MS-T0 and MS-T1 and they were therefore assigned to the persistent RRMS-associated pattern despite the DMT (Figure 3a). Dihydrolipoyl dehydrogenase (P09622), although found with increased levels compared to HC at both time points, showed a further significant increased level in MS-T1 and was consequently not included in this persistent pattern.

Thirty-eight proteins were assigned to the baseline-attenuation pattern. They differed significantly between MS-T0 and HC, changed significantly between MS-T0 and MS-T1 towards the HC abundance level, and no longer differed significantly from HC at MS-T1 (Figure 3b). Among these, 26 proteins showed increased levels in MS-T0 compared to HC and decreased level in MS-T1, whereas 12 showed the opposite profile. Proteins belonging to the baseline-attenuation pattern showed directional concordance between HC-based and longitudinal comparisons; proteins increased in MS-T0 compared to HC were generally decreased after DMT, whereas proteins decreased in MS-T0 compared to HC were increased in MS-T1.

Twenty-five proteins were assigned to the treatment-associated pattern that identified protein differences that became detectable only after six months of DMT. These proteins did not differ significantly between MS-T0 and HC but became significantly different from HC at MS-T1 (Figure 3c). Eleven proteins showed increased abundance and 14 showed decreased abundance in MS-T1 compared to HC.

**Figure 4:**
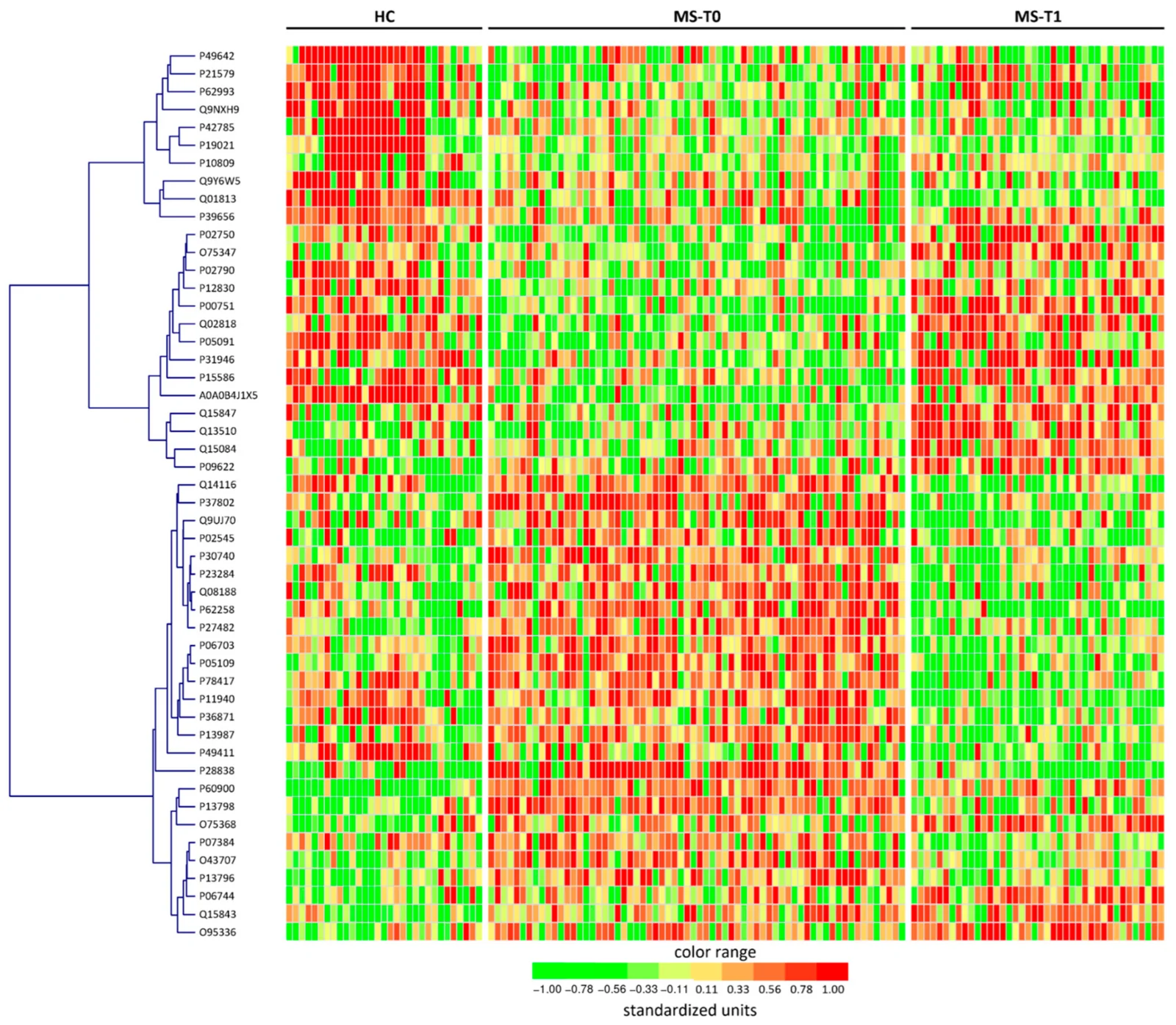
Heatmap showing the abundance profiles of the 50 proteins selected by the Boruta feature selection algorithm across HC, MS-T0 and MS-T1. Protein abundances were row-wise standardized (mean = 0, SD = 1) and grouped using hierarchical clustering. Colors indicate relative protein abundance, ranging from low (green) to high (red). Distinct abundance patterns can be observed among the three groups, including proteins persistently altered in RRMS, proteins displaying shifts in levels towards those of HC following treatment, and proteins exhibiting treatment-associated abundance patterns. These different patterns are also displayed by the dendrogram clusters.

An analogous proteomics patterns profile was also evidenced in the heatmap generated using the 50 proteins selected by the Boruta algorithm for RF classification (Figure 4). Despite the smaller number of proteins, the set of proteins selected by Boruta retained examples of the three main abundance patterns among HC, MS-T0 and MS-T1 groups corresponding to the persistent RRMS-associated, baseline-attenuation and treatment-associated patterns identified through the pairwise comparisons.

In addition to these three patterns, analysis of the paired MS-T0 and MS-T1 samples identified a longitudinal-only proteomic remodelling (Figure 3d). This comprised 36 proteins showing significant variation during the six-month follow-up without reaching statistical significance in the comparisons with HC at either time point. Of these, 17 proteins increased and 19 decreased in MS-T1 compared to MS-T0.

### 3.5. Functional Characterization of Persistent, Baseline-Attenuation and Treatment-Associated Signatures

Reactome enrichment analyses were performed to characterize the biological processes associated with the main patterns of protein sets identified among HC, MS-T0 and MS-T1 groups by separating proteins with significantly increased and decreased levels for each analysis.

No significant pathways after FDR correction were found for the proteins included in the persistent RRMS-associated signature; however, their biological functions highlighted involvement in carbohydrate and energy metabolism, such as Fructose-bisphosphate aldolase A (P04075), Triosephosphate isomerase (P60174) and L-lactate dehydrogenase A chain (P00338), together with involvement in redox homeostasis and innate immune-related processes, including Glutathione S-transferase P (P09211), Thioredoxin (P10599) and Lactoperoxidase (P22079). Proteostasis- and protein-turnover-related components included Proteasome subunit alpha type-6 (P60900), NEDD8 (Q15843), 10 kDa heat shock protein, mitochondrial (P61604) and Calpastatin (P20810), all persistently increased in patients at both time points. Proteins linked to cytoskeletal organization and extracellular remodelling included Tropomyosin alpha-4 chain (P67936), Annexin A2 (P07355) and Gelsolin (P06396), which were persistently increased, together with Cytoplasmic dynein 1 heavy chain 1 (Q14204), which was decreased. The complete list of protein pertaining to this signature is reported in Supplementary Table S4a.

Reactome enrichment analysis of the baseline-attenuation signature revealed significant enrichment of immune- and cell-death-related pathways, including Neutrophil degranulation, Innate Immune System, Immune System, Apoptosis, Programmed Cell Death, Apoptotic execution phase and Apoptosis induced DNA fragmentation. These pathways were predominantly supported by proteins decreased in MS-T1 compared to MS-T0, specifically, proteins with increased levels at baseline but trending towards HC levels following treatment.

**Figure 5:**
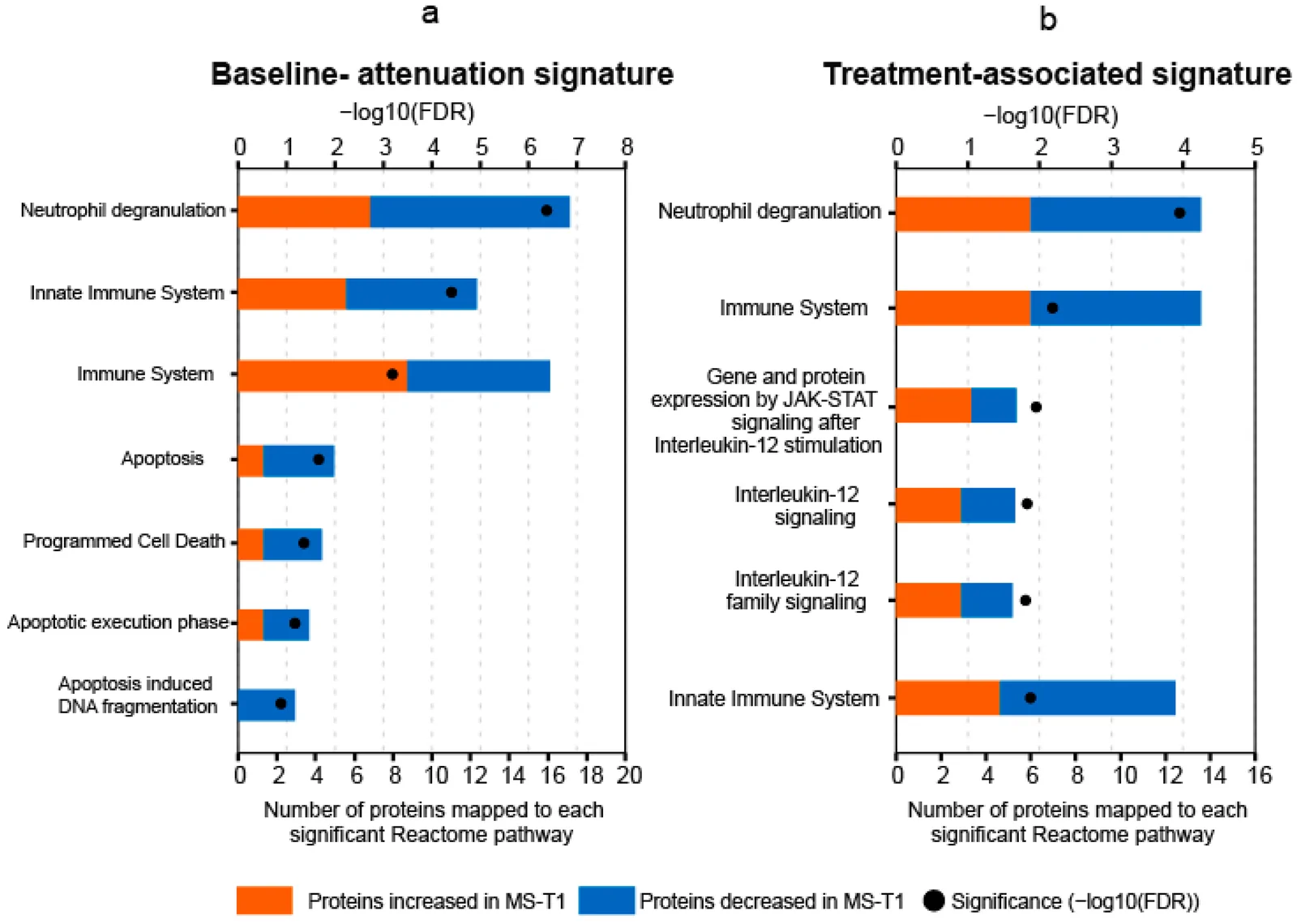
Functional characterization of baseline-attenuation and treatment-associated proteomic patterns. (**a**) Reactome pathway enrichment analysis performed on proteins of the baseline-attenuation signature. Significantly enriched pathways are represented as stacked horizontal bars, with the total number of mapped proteins partitioned according to whether proteins decreased or increased in MS-T1 compared to MS-T0. (**b**) Reactome pathway enrichment analysis of the treatment-associated signature. For each pathway, the total number of mapped proteins is partitioned according to whether proteins were increased or decreased in MS-T1 compared to HC. Black dots indicate pathway significance expressed as −log10(FDR-adjusted *p*-value).

Representative examples included Protein S100-A8 (P05109), Protein S100-A9 (P06703), Azurocidin (P20160), High mobility group protein B1 (P09429), Proteasome subunit beta type-1 (P20618) and Leukocyte elastase inhibitor (P30740). A smaller contribution was provided by proteins increased in MS-T1, including Leucine-rich alpha-2-glycoprotein (P02750), Leukotriene A-4 hydrolase (P09960) and Cadherin-1 (P12830). The complete list of enriched pathways and the proteins involved is reported in Supplementary Table S4b, while their directional contribution is illustrated in Figure 5a.

Reactome enrichment analysis of the treatment-associated signature revealed a targeted enrichment of immune system-related pathways, including Neutrophil degranulation, Immune System, Innate Immune System, with a contribution of protein expressed by JAK–STAT signalling after Interleukin-12 (IL-12) stimulation and IL-12 family signalling. Neutrophil degranulation and Innate Immune System were mainly supported by proteins decreased in MS-T1, whereas IL-12-related pathways showed a smaller and mixed contribution, with a predominance of proteins increased in MS-T1. Representative proteins included Acid ceramidase (Q13510), Glucose-6-phosphate isomerase (P06744), Superoxide dismutase [Cu-Zn] (P00441), Resistin (Q9HD89) and Glutathione S-transferase omega-1 (P78417). The complete list of enriched pathways and involved proteins is reported in Supplementary Table S4c, while their directional contribution is summarized in Figure 5b.

### 3.6. Longitudinal Proteomic Changes Following DMT

To further investigate proteomic changes associated with DMT, salivary protein abundances were compared between MS-T0 and MS-T1. Since only a subset of patients returned for the follow-up sampling, both unpaired and paired analyses were performed. The unpaired comparison included all available MS-T0 (n = 66) and MS-T1 (n = 40) samples, whereas the paired analysis was restricted to the 40 patients sampled at both time points.

The unpaired comparison identified 120 differentially abundant proteins, whereas the paired analysis identified 100 proteins showing significant longitudinal variation. In both analyses, proteins with reduced abundance in MS-T1 slightly predominated, accounting for 61/120 proteins in the unpaired analysis and 60/100 proteins in the paired analysis.

**Figure 6:**
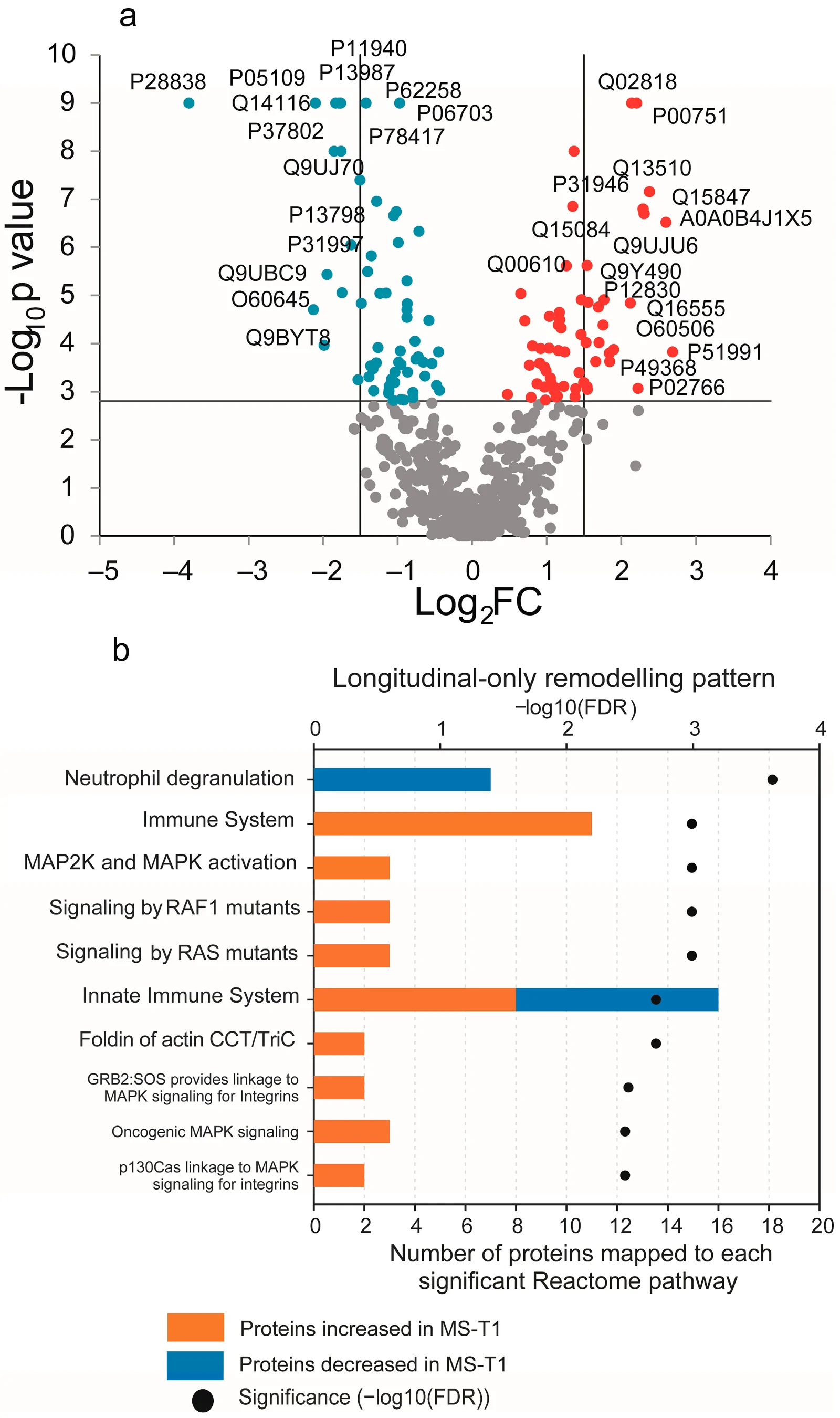
Longitudinal proteomic changes associated with DMT. (**a**) Volcano plot of the unpaired comparison between all available MS-T0 (n = 66) and MS-T1 (n = 40) samples. Significantly increased proteins are shown in red, significantly decreased proteins in blue and all others in grey. The horizontal line at -Log10 *p*-value = 2.8 corresponds to the significant *p*-values threshold value (*p* < 0.001552, with an FDR < 1%). The vertical lines correspond to the Log2 FC threshold of <−1.5 or >1.5. Only the proteins falling within these *p*-value and FC ranges are labelled. (**b**) Significantly enriched Reactome pathways identified from longitudinal-only proteins changing between MS-T0 and MS-T1 in the paired analysis are shown as horizontal bars. Orange bars indicate pathways enriched among longitudinal-only proteins increased in MS-T1 compared to MS-T0, whereas blue bars indicate pathways enriched among longitudinal-only proteins decreased in MS-T1 compared to MS-T0. For pathways enriched in both directional protein sets, orange and blue segments are shown within the same stacked bar. Black dots indicate pathway significance expressed as −log10(FDR-adjusted *p*-value).

The volcano plot of the unpaired MS-T1 versus MS-T0 comparison (Figure 6a) illustrates the distribution of longitudinal protein changes after six months of DMT. Among the proteins showing the strongest decrease level in MS-T1 were Cytosol aminopeptidase (P28838), Protein S100-A8 (P05109), 14-3-3 protein epsilon (P62258), Protein S100-A9 (P06703) and Acylamino-acid-releasing enzyme (P13798). These proteins presented increased or decreased levels in the MS-T0 group compared to HC and tended towards HC levels following DMT, supporting their inclusion in the baseline-attenuation signature. Polyadenylate-binding protein 1 (P11940) was also among the proteins with strong reduced level in MS-T1, but it fell within the treatment-associated signature as its decrease became significant only following exposure to the therapy.

Conversely, proteins showing increased abundance in MS-T1 relative to MS-T0 included Nucleobindin-1 (Q02818), Complement factor B (P00751), Acid ceramidase (Q13510), Adipogenesis regulatory factor (Q15847), 14-3-3 protein beta/alpha (P31946), Immunoglobulin heavy variable 3-74 (A0A0B4J1X5), Protein disulfide-isomerase A6 (Q15084), 14-3-3 protein eta (Q04917) and UMP-CMP kinase (P30085). This group included proteins with different patterns: some were reduced at MS-T0 compared to HC and increased after treatment, consistent with a baseline-attenuation profile; others became significantly altered only at MS-T1, supporting treatment-associated proteomic remodelling; and a further subset changed only between paired MS-T0 and MS-T1 samples, contributing to the longitudinal-only remodelling signature.

Among the longitudinally-only modulated proteins, mitochondrial Dihydrolipoyl dehydrogenase (P09622) represented a notable example of a persistent RRMS-associated alteration with additional longitudinal modulation. This protein was significantly increased in both MS-T0 and MS-T1 groups compared to HC but showed a further increase in MS-T1 with respect to MS-T0.

To specifically characterize the longitudinal-only remodelling signature, exploratory Reactome enrichment analysis was performed separately on proteins increased and decreased in MS-T1 compared to MS-T0 in the paired analysis (Figure 6b). Proteins increased in MS-T1 were enriched in immune-related pathways, including Immune System and Innate Immune System, together with MAPK/RAF/RAS-related signalling, integrin-associated MAPK signalling and CCT/TriC-mediated protein folding. Representative proteins included 14-3-3 protein beta/alpha (P31946), Talin-1 (Q9Y490), Fibrinogen alpha chain (P02671) and T-complex protein 1 subunit beta (P78371). Conversely, proteins decreased in MS-T1 showed a more restricted enrichment profile, mainly involving Neutrophil degranulation and Innate Immune System, with representative proteins including Carcinoembryonic antigen-related cell adhesion molecule 8 (P31997), Galectin-3 (P17931), Bactericidal permeability-increasing protein (P17213) and Integrin alpha-M (P11215). The complete list of enriched pathways and contributing proteins is reported in Supplementary Table S4d,e.

## 4. Discussion

The present study provides a comprehensive proteomic characterization of WS from patients with RRMS, showing that the salivary proteome differs between patients and HC and undergoes substantial change after six months of DMT. By integrating differential abundance analysis, exploratory machine-learning classification, Reactome pathway enrichment, and longitudinal analysis, we identified multiple layers of salivary proteomic variation. Importantly, the proteomic profile observed after DMT exposure did not correspond to a complete restoration of the healthy state. Instead, selected baseline differences shifted towards HC abundance levels, while other alterations persisted or became detectable only at MS-T1 without establishing a pharmacological treatment effect, treatment-specific mechanisms, or clinical response.

Although proteomic studies in RRMS have predominantly focused on CSF and blood, a previous study of the salivary proteome in RRMS showed that saliva can reveal disease-associated molecular differences between patients and HC, supporting its investigation as a non-invasive biofluid for biomarker discovery [13]. WS contains proteins originating from salivary gland secretions, oral mucosal surfaces, resident and recruited immune cells, extracellular vesicles, and blood-derived components reaching the oral cavity [8,9,12]. Its proteomic composition may therefore reflect molecular changes associated with both oral and systemic conditions [10,11,13].

### 4.1. Salivary Proteomics Revealed RRMS-Associated Alterations

Salivary proteomic alterations were more extensive before therapy initiation than after six months of DMT, as shown by the higher number of differentially abundant proteins in MS-T0 than in MS-T1 compared to HC. This finding indicates that the salivary proteome of RRMS patients sampled before initiation DMT exhibits a detectable RRMS-associated molecular signature. Specifically, the reduction in the number of proteins differing from HC after therapy suggests partial attenuation of the baseline alteration.

The persistent RRMS signature identified in the present study is particularly relevant because it included proteins altered in the same direction in both MS-T0 and MS-T1 compared to HC, without significant variation between the two RRMS time points. The protein composition of this persistent signature suggests their involvement in metabolic regulation, redox homeostasis, proteostasis, cytoskeletal remodelling and mucosal/innate defence.

Several proteins in this signature are enzymes of carbohydrate and energy metabolism, including Fructose-bisphosphate aldolase A, Triosephosphate isomerase and L-lactate dehydrogenase A chain. Recent serum proteomic profiling of untreated patients with MS also identified glycolysis, gluconeogenesis and glucose-metabolism pathways among the processes distinguishing MS clinical phenotypes, supporting the contribution of metabolic remodelling to the broader molecular landscape of the disease [22].

Other proteins of the persistent signature were involved in redox regulation and oxidative stress responses, such as Glutathione S-transferase P and Thioredoxin. Altered salivary antioxidant capacity has also recently been reported in RRMS, supporting the possibility that saliva may reflect redox-related changes associated with the disease [23]. The persistence of redox-related proteins in both MS-T0 and MS-T1 might therefore reflect a residual salivary redox imbalance or a compensatory antioxidant response that has not fully normalized after six months of DMT.

Proteins linked to innate defense, extracellular remodelling and mucosal homeostasis represented a further component of the persistent signature and included Annexin A2, Gelsolin and Lactoperoxidase. Annexin A2 has been implicated in blood–brain barrier-related mechanisms in experimental autoimmune encephalomyelitis, a mouse model of multiple sclerosis [24], whereas gelsolin has been studied in CSF of RRMS patients and experimental neuroinflammation in relation to actin scavenging and inflammatory modulation [25,26]. Lactoperoxidase (LPO) is a key antimicrobial component of the salivary innate defence. Increased levels of LPO have been reported in the tear fluid of patients with multiple sclerosis, although in a small exploratory study [27], and also in cerebrospinal fluid in Parkinson’s disease [28] and, more recently, LPO has shown to be selectively expressed in human dopaminergic neurons of the substantia nigra [29]. Even if LPO is not restricted to salivary secretion, the salivary alteration we observed may be related primarily to mucosal or peripheral inflammatory processes persisting after the six months of DMT and does not establish its involvement in central nervous system pathology in RRMS.

### 4.2. Machine-Learning Signature Reveals Biologically Coherent Immune-Related Salivary Patterns

The RF analysis combined with Boruta feature selection further supported the discriminative value of the salivary proteome. Specifically, the set of 50 proteins selected by Boruta achieved an OOB classification accuracy of 95.4%, indicating strong internal discrimination among HC, MS-T0 and MS-T1 samples and supporting that group separation was driven by a coordinated multivariate protein pattern. This aspect is particularly relevant in high-dimensional proteomic datasets, where feature selection allows to reduce background noise and highlight the proteins that jointly contribute to classification. The contribution of 14-3-3 protein epsilon, which showed high class-specific importance despite not being among the strongest univariate markers, further supports the value of multivariate approaches in highlighting coordinated proteomic relationships that may be missed by single-comparison analyses.

Related strategies that combine mass spectrometry and machine learning analyses have been successfully used in our previous works evidencing alteration on the salivary proteome in patients with neurodegenerative and immunological diseases [11,30,31]. Nevertheless, since the OOB classification accuracy was obtained through internal validation within our cohort, it requires confirmation in independent samples. These 50 proteins therefore represent a candidate biomarker panel that could form the basis of a predefined model to be evaluated in an independent cohort, ideally including longitudinal sampling and different DMTs.

Among the most informative proteins, ATP-dependent 6-phosphofructokinase, platelet type, Cytosol aminopeptidase, Polyadenylate-binding protein 1, Proteasome subunit alpha type-6 and 14-3-3 protein epsilon contributed to the discrimination of HC, MS-T0 and MS-T1 samples. These proteins are functionally heterogeneous, involving metabolic regulation, proteolysis, protein homeostasis and cellular stress-related processes. ATP-dependent 6-phosphofructokinase, platelet type, for example, is a glycolytic enzyme, and altered immunometabolism has been described in RRMS, including impaired glycolysis and mitochondrial respiration in T cells from patients with RRMS [32].

Importantly, Reactome enrichment analysis performed on the 50 proteins selected by Boruta showed significant enrichment of Neutrophil degranulation, Innate Immune System and Immune System. This indicates that the salivary protein signature evidenced by the machine-learning approach was not only statistically discriminant, but also biologically coherent with the immune-related processes emerging from the pattern-based analyses and known to be implicated in multiple sclerosis pathophysiology.

### 4.3. DMT Treatment Is Associated with Partial Attenuation of Baseline Inflammatory Signatures and Treatment-Associated Salivary Remodelling

Among the comparisons involving both MS-T0 and MS-T1 patients with respect to HC two complementary patterns were particularly informative. The baseline-attenuation signature comprised proteins varied in MS-T0 with respect to HC that shifted towards HC levels after six months of DMT; conversely, the therapy-associated signature included proteins with levels not significantly different in MS-T0 but exhibiting different abundances in MS-T1. This distinction is important as the first pattern suggests a partial attenuation of baseline RRMS-related alterations, whereas the second reflects the establishment of a post-DMT salivary proteomic state.

The baseline-attenuation pattern was enriched in Neutrophil degranulation, Innate Immune System, Immune System, Apoptosis, Programmed Cell Death, Apoptotic execution phase and Apoptosis-induced DNA fragmentation. This enrichment is biologically plausible in the context of RRMS, as increasing evidence indicates that neutrophils can contribute to neuroinflammatory mechanisms through priming, degranulation, oxidative burst, extracellular trap formation and modulation of blood–brain barrier permeability [33,34]. In our salivary dataset, Protein S100-A8, Protein S100-A9, Azurocidin and Leukocyte elastase inhibitor contributed to this pattern. Their decreased abundance in MS-T1 relative to MS-T0 is consistent with partial attenuation of a baseline salivary inflammatory pattern after six months of DMT exposure. Protein S100-A8 and Protein S100-A9 are calcium-binding proteins associated with innate immune activation and inflammatory danger signalling [35]. Experimental evidence indicates that the S100A8/A9 complex can promote microglial activation and oligodendrocyte precursor-cell stress through nuclear factor kappa B (NF-κB)-mediated mechanisms [35].

Consistent with these findings, a previous top-down salivary proteomic study detected increased levels of an S-nitrosylated Protein S100-A8 proteoform in patients with MS, particularly in those not receiving DMT [13]. Although that cross-sectional observation cannot be interpreted as a longitudinal treatment effect, it is conceptually consistent with the DMT-associated partial-convergence pattern identified here.

Concordance is also evident at the functional level in proteomic studies performed in other biological compartments. Reactome analysis of serum proteins from untreated patients with MS identified enrichment of Innate Immune System and Neutrophil degranulation, together with cellular responses to oxidative stress and protein-metabolism pathways [22]. Similarly, a small longitudinal plasma multi-omics study associated magnetic resonance imaging activity with innate inflammatory responses and neutrophil degranulation, although complement and coagulation pathways were also prominent [36]. At the transcriptomic level, RNA sequencing of peripheral-blood neutrophils identified neutrophil degranulation as the most significantly enriched process among upregulated genes, including LTF, ELANE, AZU1 and BPI [33]. These observations provide independent support for the involvement of neutrophil-related and innate-immune processes in MS. Taken together, these observations indicate that the salivary proteome, despite its distinct composition, reflects at the pathway level immune-related molecular features independently identified in other peripheral matrices.

High mobility group protein B1, another protein decreased in MS-T1 within the baseline-attenuation signature, has also been investigated as a circulating marker of inflammatory activity in MS [37]. In agreement with our data, lower circulating levels of this protein have been reported in RRMS patients following DMT although this was in a cross-sectional study that did not specifically evaluate a six-month treatment interval [38]. It is noteworthy that a similar partial attenuation of baseline RRMS-related alterations after few months of treatment was reported by Huang et al., who found that three to six months of natalizumab treatment reduced several multiple sclerosis-associated inflammatory proteins in CSF, narrowing their differences from healthy controls [39]. The same study also highlighted differences among the different DMTs, with fingolimod primarily affecting plasma proteins. Collectively, our results reinforced the findings that peripheral biofluids, saliva comprised, may detect non-invasively proteomic changes following DMT, without establishing it as a direct mirror for central nervous system proteomic changes.

The treatment-associated signature was characterized by proteins involved in pathways enriched in Neutrophil degranulation, Immune System, Innate Immune System and IL-12/JAK–STAT-related pathways. These findings suggest that six months of DMT was associated not only with partial attenuation of specific alterations observed in MS-T0 but also with the appearance of a distinct post-treatment salivary molecular profile in MS-T1. The immune-related enrichment was directionally heterogeneous. Neutrophil degranulation and Innate Immune System were predominantly supported by proteins with decreased abundance in MS-T1, whereas the IL-12/JAK–STAT-related annotations were supported by the same small set of three proteins that varied in opposite direction: Superoxide dismutase and S-methyl-5-thioadenosine phosphorylase which increased, and Glutathione S-transferase omega-1 which decreased in MS-T1, supporting the hypothesis of a protein pattern remodelling. The IL-12/JAK–STAT-related annotations are biologically plausible in view of genetic and functional evidence linking IL-12/STAT signalling to immune-cell responses in MS [40]. Consistently, a large-scale plasma proteomic study reported suggestive enrichment of JAK–STAT signalling in individuals with MS [41].

Other representative proteins in this signature included Acid ceramidase, Glucose-6-phosphate isomerase, Superoxide dismutase [Cu-Zn], Resistin and Glutathione S-transferase omega-1, which are notoriously involved with biological axes related to lysosomal sphingolipid turnover, metabolic regulation, oxidative stress responses and inflammatory adipokine-mediated signalling.

The evidence on Acid ceramidase is of potential interest because altered ceramide metabolism has been reported in RRMS [42], although sphingolipid metabolism was not significantly enriched in the present dataset. Resistin and other adipokines have also been investigated in relation to immune regulation in RRMS [43], while salivary and plasma oxidative-stress markers have previously been reported in multiple sclerosis [23].

Exploratory analyses aimed to assess drug-specific effects did not identify robust differences after correction for multiple testing, either when comparing teriflunomide versus dimethyl fumarate or when grouping treatments into first-line versus high-efficacy DMT. Similarly, no pattern-level profile remained significantly associated with the treatment subgroup after FDR correction. Therefore, the treatment-related signature should be interpreted as a molecular profile emerging following DMT in the overall cohort.

### 4.4. Longitudinal-Only Remodelling Reveals Intra-Individual Salivary Changes

An additional layer of information was provided by the longitudinal-only remodelling signature, comprising proteins that changed significantly between paired MS-T0 and MS-T1 samples without showing significant differences compared to HC at either time point. This pattern is particularly relevant because it would not have been revealed by cross-sectional comparisons alone.

Longitudinal-only proteins with increased levels in MS-T1 were enriched in immune-related pathways, MAPK/RAF/RAS-related signalling, integrin-associated MAPK signalling and CCT/TRiC-mediated protein folding.

The presence of MAPK-related annotations is biologically plausible in RRMS, since alteration of this pathway has already been found implicated in neuroinflammatory mechanisms related to RRMS [44]. In particular, increased serum levels of Talin-1 have been reported in RRMS patients and are associated with disease activity [45], while autoantibodies against Talin-1 have also been described in RRMS sera [46]. The fibrinogen alpha chain is biologically relevant in the broader context of coagulation, blood–brain barrier dysfunction and inflammatory tissue remodelling, as fibrinogen/fibrin deposition has been implicated in CNS autoimmunity, demyelination and innate immune activation in RRMS and experimental models [36,47].

Similarly, the enrichment of CCT/TRiC-mediated protein folding, supported by the increased abundance of T-complex protein 1 subunit beta and subunit gamma in MS-T1, suggests longitudinal variation in protein-homeostasis mechanisms after DMT. The CCT/TRiC complex is an ATP-dependent cytosolic chaperonin involved in the folding of several cytoskeletal and regulatory proteins, including actin and tubulin-related substrates, and contributes to cellular proteostasis [48]. Recent evidence has linked the CCT complex, particularly CCT3, to ANKRD55-dependent regulation of immune synapse formation and TCR activation in T-cell inflammation [49].

Conversely, longitudinal-only proteins with decreased levels in MS-T1 showed a more restricted enrichment profile, mainly involving Neutrophil degranulation and the Innate Immune System. Representative proteins included Galectin-3, Bactericidal permeability-increasing protein, Integrin alpha-M and Carcinoembryonic antigen-related cell adhesion molecule-8. Of note, Bactericidal permeability-increasing protein was also included among the neutrophil-degranulation-related genes identified as upregulated by peripheral-blood neutrophil transcriptomics in MS [33], providing additional support for pathway-level convergence involving neutrophil-related processes. Taken together, the proteins found increased and decreased in the longitudinal comparison highlighted that the salivary proteome did not change uniformly over the six months of DMT. Instead, distinct groups of proteins changed in opposite directions and were associated with different biological processes. Therefore, the identified proteins may probably reflect intra-individual temporal remodelling occurring during the six-month follow-up but cannot be interpreted as either restoration towards or divergence from the HC profile.

### 4.5. Limitations

Although the MS-T0 cohort included 66 RRMS patients, follow-up saliva samples were available for only 40 patients, thereby limiting the statistical power of paired longitudinal analyses.

The machine-learning analysis was exploratory. RF accuracy, although very high, was validated only by out-of-bag samples, and not by external, independent datasets, and this represents a major risk of overfitting.

Although age did not differ significantly among the study groups, healthy controls were numerically younger than patients with RRMS; therefore, residual age-related confounding cannot be excluded, and age may have contributed to some of the observed differences in salivary protein abundance. Future studies should confirm these findings in independently recruited age-matched cohorts.

Previous treatment exposure represents an additional limitation. Although previous therapies were discontinued at least one month before sampling and patients switching from teriflunomide underwent accelerated elimination, pharmacokinetic clearance does not necessarily coincide with the resolution of pharmacodynamic effects. Residual biological effects of previous treatments may therefore have influenced the baseline salivary proteome and contributed to longitudinal changes, limiting their attribution solely to the newly initiated DMT.

Since patients received different treatments and the individual treatment subgroups were small, the study lacked the statistical relevance to support drug-specific conclusions.

The absence of an untreated longitudinal RRMS group prevents separation of changes associated with DMT exposure from those related to disease evolution or other time-varying influences. Although the paired design controls for stable participant-level characteristics and the same standardized collection protocol were applied at both visits, oral and systemic conditions were not documented comprehensively and symmetrically at T0 and T1 and may therefore represent residual confounders. Larger multicenter longitudinal studies integrating salivary analysis with clinical, radiological, and additional fluid biomarker data will be needed to validate the biological and clinical relevance of the proposed salivary signatures.

## 5. Conclusions

Overall, this study supports whole saliva as a promising, non-invasive biofluid for longitudinal molecular monitoring in RRMS. By combining high-resolution mass spectrometry, differential abundance analysis, machine-learning-based classification and paired longitudinal analysis, we identified a salivary proteomic signature that distinguished RRMS patients from HC and evidenced molecular remodelling associated with six months of DMT.

The panel of 50 proteins selected by RF/Boruta distinguished HC, MS-T0 and MS-T1 samples with an overall out-of-bag accuracy of 95.4% within the present cohort. Moreover, 48 of the 50 proteins (96%) also exhibited significant changes in univariate tests. Thus, within the limitations remarked in the previous section, the 50-protein panel could be considered as a candidate proteomic signature of the RRMS panel that could form the basis of a predefined model to be evaluated in an independent cohort.

Among the highest-ranked features, ATP-dependent 6-phosphofructokinase, platelet type and Proteasome subunit alpha type-6 remained, respectively, decreased and increased at both RRMS time points. Conversely, Cytosol aminopeptidase, 14-3-3 protein epsilon and Protein S100-A8 shifted towards HC levels after six months of DMT and may be more suitable for longitudinal monitoring. Polyadenylate-binding protein 1, which became altered only at MS-T1, may instead reflect the post-treatment salivary proteomic state. At the functional level, these patterns indicated partial attenuation of neutrophil- and innate immunity-related alterations together with the emergence of distinct immune remodelling at MS-T1.

Finally, it is worth noting that the present study represents a preliminary, exploratory investigation of the RRMS proteome, so the results obtained, although of some interest, are not suitable for diagnostic applications or clinical evaluations.

## Figures and Tables

**Table 1:** Demographic and clinical characteristics of RRMS patients and HC subjects included in the salivary proteomics analysis. MS-T0 and MS-T1 refers to samples collected before and after six months of therapy, respectively.

	MS-T0	MS-T1	HC
Number of subjects/samples, n	66	40	31
Female sex, n (%)	46 (69.7%)	29 (72.5%)	23 (74.2%)
Protein conc. µg/µL, mean ± SD	1.7 ± 0.8	1.7 ± 0.8	1.4 ± 0.4
Age at baseline, years, mean ± SD	41.6 ± 11.5	43.5 ± 11.5	38.4 ± 12.8
Age at baseline, years, median (IQR)	40.5 (32.0–47.5)	43.0 (32.8–55.3)	33.0 (28.5–47.0)
Age at baseline, years, range	20–67	26–63	23–69
Age at onset, years, mean ± SD	38.8 ± 11.7		
Age at onset, years, median (IQR)	36.0 (30.0–46.0)		
Disease duration at baseline, years, mean ± SD	3.9 ± 3.9		
Disease duration at baseline, years, median (IQR)	2.0 (1.0–7.0)		
Treatment-naïve at baseline, n (%)	18 (27.3%)		
Previously treated at baseline, n (%)	48 (72.7%)		
DMT assigned after baseline, n (%)	Teriflunomide: 18 (27.3%); Dimethyl fumarate: 18 (27.3%); Cladribine: 12 (18.2%); Ocrelizumab: 12 (18.2%); Natalizumab: 4 (6.1%); Ozanimod: 2 (3.0%)		
EDSS at baseline, mean ± SD	2.4 ± 0.9		
EDSS at baseline, median (IQR)	2.0 (2.0–3.0)		
EDSS at baseline, range	1.0–5.0		
MRI activity at baseline, n (%)	43 (65.2)		

EDSS: Expanded Disability Status Scale; HCs: Healthy Controls; IQR: interquartile range; MRI: Magnetic Resonance Imaging; SD: Standard Deviation.

## Data Availability

The mass spectrometry proteomics data has been deposited with the ProteomeXchange Consortium via the PRIDE partner repository under dataset identifier PXD053357.

## Supplementary Materials

The following supporting information can be downloaded at: https://www.mdpi.com/article/10.3390/biom16101524/s1, Figure S1: Illustrative classification tree generated within the RF outputs. For visualization purposes, the tree was constructed using proteins emerged as the most important in the classification according to Mean Decrease Accuracy (MDA); Internal nodes show UniProt accession codes and split points for the processed protein abundance values; the corresponding protein names are reported in Supplementary Table S3. Terminal nodes indicate the predicted classes. “Y” indicates that the condition specified at the node is true, whereas “N” indicates that it is not true. In the standard RF procedure, each tree is randomly generated using only a limited number of variables and two-thirds of subjects. Samples not included contribute to estimation of the out-of-bag error. Some proteins may therefore appear at different split points within a tree, while other trees (compressively 1500) may use different proteins in the split points. This explains the conditional, nonlinear and multivariate nature of RF classification and helps explain why the results of the RF occasionally diverge from those of univariate tests. HC, healthy controls; MS-T0, patients with relapsing-remitting multiple sclerosis at baseline; MS-T1, patients after six months of disease-modifying therapy; Figure S2: Heatmap of the 209 proteins with significant abundance differences among HC, MS-T0 and MS-T1. Protein abundances were row-wise standardized (mean = 0, SD = 1) and clustered using hierarchical clustering, to obtain homogeneous subsets. Colors represent relative protein abundance, ranging from low (green) to high abundance (red); Table S1a: Clinical and demographic characteristics of MS-T0 patients included in the proteomic analyses. Switchers are patients transitioning from first-line therapies with these treatments discontinued for at least one month at the time of enrollment. Patients reporting DMT at follow-up represent the MS-T1 cohort used for the longitudinal study; Table S1b: Demographic characteristics of HC subjects included in the proteomic analyses; Table S1c: Comparison of MS-T0 characteristics between follow-up completers and non-completers; Table S1d: Sensitivity analysis comparing treatment-naïve and previously treated (switcher) patients at MS-T0; Table S2a: Wilcoxon rank-sum test comparisons between the three groups: MS-T0, MS-T1, and HC. Paired comparions between MS-T0 and MS-T1 by the Wilcoxon signed rank test are also shown (last column). For each comparison the significant *p*-value with a 1% FDR (cut-off 0.00165) and the direction of the change are reported. Zero values indicate *p*-values < 0.0001; Table S2b: Spearman rank correlations were calculated between each clinical or demographic variable and the abundance of each of the 623 proteins, separately at baseline (MS-T0), six months after disease-modifying therapy exposure (MS-T1), and for the paired abundance changes between the two time points. Benjamini–Hochberg correction was applied separately within each combination. The last two columns report the number of proteins with a nominal *p*-value < 0.05 and the number remaining significant at a false discovery rate < 0.05, respectively; Table S2c: FDR-significant associations between salivary protein abundance at MS-T0 and age-related clinical variables; Table S3: Relative importance of proteins for Random Forest classification. The table reports the Mean Decrease Accuracy (MDA) of the 50 proteins selected by the Boruta algorithm for RF. The MDA reflects the reduction in classification accuracy observed when the values of a given protein are randomly permuted; therefore, higher values indicate a stronger contribution of that protein to the classification. The MDA index is calculated for the overall classification model (global, in decreasing order) and separately for each class: HC, MS-T0, and MS-T1; Table S4a: Persistent RRMS signature: protein composition; Table S4b: Baseline-attenuation signature: significant Reactome pathways and contributing proteins; Table S4c: Treatment-associated signature: significant Reactome pathways and contributing proteins; Table S4d: Longitudinal-only remodeling signature: proteins increased in MS-T1; Table S4e: Longitudinal-only remodeling signature: proteins decreased in MS-T1; Supplementary File S1: Computational environment, preprocessing workflow and parameters used for Boruta feature selection and Random Forest classification; Video S1: Three-dimensional multidimensional-scaling visualization of the Random Forest proximity matrix.
