# Supplementary material for: Salivary Proteomics Reveals Disease- and Therapy-Associated Molecular Patterns in Relapsing-Remitting Multiple Sclerosis
Source: Biomolecules. 2026 Oct 19;16(10):1524. doi: 10.3390/biom16101524 (PMC13702458; doi:10.3390/biom16101524)
Supplement: Supplementary file 1 [file biomolecules-16-01524-s001.zip › Supplematary File S1.pdf]

Supplementary File S1. Computational environment, preprocessing workflow and parameters used for Boruta feature selection and Random Forest classification.

Analyses were performed in R version 4.4.2 using a random seed of 7. Boruta version 9.0.0 was run with `maxRuns = 100` (default setting); proteins classified as Confirmed were retained, whereas Tentative and Rejected features were excluded. Random Forest classification was performed using the "RandomForest" package version 4.7-1.2, with 1500 trees (`ntree = 1500`) and 10 variables randomly sampled at each split (`mtry = 10`, selected after tuning). The original group sizes were 31 healthy controls, 66 MS-T0 samples and 40 MS-T1 samples. Class balancing was implemented by sampling 31 observations from each group for each tree (`sampsize = 31, 31, 31`), with equal class weights (`classwt = 1, 1, 1`). Preprocessing was applied to the complete dataset before feature selection and model fitting and comprised, in sequence: (1) log10 transformation; (2) imputation of left-censored missing values using the quantile-regression-based `imputeQRILC` method implemented in the `imputeLCMD` package; and (3) quantile normalization.
