# Supplementary material for: Salivary Proteomics Reveals Disease- and Therapy-Associated Molecular Patterns in Relapsing-Remitting Multiple Sclerosis
Source: Biomolecules. 2026 Oct 19;16(10):1524. doi: 10.3390/biom16101524 (PMC13702458; doi:10.3390/biom16101524)
Supplement: Supplementary file 1 [file biomolecules-16-01524-s001.zip › supplemental - REV1.pdf]

Supplementary Table S1a. Clinical and demographic characteristics of MS-T0 patients included in the proteomic analyses. Switchers are patients transitioning from first-line therapies with these treatments discontinued for at least one month at the time of enrollment. Patients reporting DMT at follow-up represent the MS-T1 cohort used for the longitudinal study.

T0 code	Age	Sex	Age at baseline	Age at onset	Naïve/ Switcher	EDSS at baseline	DMT at follow-up
**BL01**	57	F	58	57	Naïve	2,5	Teriflunomide
**BL02**	59	F	60	59	Naïve	3,0	Teriflunomide
**BL03**	62	F	63	62	Naïve	3,5	Teriflunomide
**BL04**	43	F	44	37	Switcher	5,0	Teriflunomide
**BL05**	56	F	57	51	Switcher	2,5	Teriflunomide
**BL06**	37	F	38	36	Switcher	2,5	Teriflunomide
**BL07**	41	M	42	39	Switcher	4,5	Teriflunomide
**BL08**	55	M	56	49	Switcher	3,5	Teriflunomide
**BL09**	63	F	64	57	Switcher	3,5	Teriflunomide
**BL10**	63	F	64	63	Switcher	4,0	Teriflunomide
**BL11**	58	F	59	59	Naïve	2,5	Teriflunomide
**BL25**	38	F	39	38	Naïve	2,5	Dimethyl fumarate
**BL26**	32	F	34	33	Switcher	2,0	Dimethyl fumarate
**BL27**	45	F	46	39	Switcher	1,5	Dimethyl fumarate
**BL28**	50	F	51	46	Switcher	1,5	Dimethyl fumarate
**BL29**	32	M	33	32	Naïve	1,0	Dimethyl fumarate
**BL30**	51	M	52	51	Naïve	1,5	Dimethyl fumarate
**BL31**	43	M	44	37	Switcher	2,5	Dimethyl fumarate
**BL32**	39	F	40	33	Switcher	2,0	Dimethyl fumarate
**BL33**	57	M	58	57	Naïve	2,0	Dimethyl fumarate
**BL34**	45	F	47	46	Naïve	2,0	Dimethyl fumarate
**BL35**	30	F	31	29	Switcher	2,0	Dimethyl fumarate
**BL36**	40	M	41	40	Switcher	3,0	Dimethyl fumarate
**BL37**	29	F	30	29	Naïve	1,0	Dimethyl fumarate
**BL12**	35	F	36	32	Switcher	2,0	Cladribine
**BL13**	59	F	61	60	Switcher	2,0	Cladribine
**BL14**	41	M	43	35	Switcher	5,0	Cladribine
**BL15**	42	F	43	36	Switcher	1,0	Cladribine
**BL16**	43	F	44	40	Switcher	1,0	Cladribine
**BL17**	27	F	29	23	Switcher	1,0	Ocrelizumab
**BL18**	28	F	30	29	Switcher	2,0	Ocrelizumab
**BL19**	60	M	61	59	Switcher	4,0	Ocrelizumab
**BL20**	31	F	32	28	Switcher	2,0	Ocrelizumab
**BL21**	43	F	44	43	Naïve	2,0	Ocrelizumab
**BL22**	43	M	44	34	Switcher	3,5	Ocrelizumab
**BL38**	29	F	30	28	Switcher	1,0	Natalizumab
**BL39**	30	F	31	30	Switcher	1,0	Natalizumab
**BL40**	26	M	27	25	Switcher	2,0	Natalizumab
**BL41**	33	F	34	33	Naïve	2,0	Ozanimod

**BL42**	44	F	45	38	Switcher	2,0	Ozanimod
**BL43**	55	F	56	55	Switcher	2,0	
**BL44**	38	M	39	35	Switcher	2,0	
**BL45**	67	M	68	67	Switcher	4,0	
**BL46**	48	F	50	49	Switcher	2,0	
**BL47**	36	M	37	26	Switcher	3,0	
**BL48**	32	M	33	33	Naïve	3,0	
**BL49**	30	F	31	30	Naïve	3,0	
**BL63**	46	M	48	33	Switcher	3,0	
**BL64**	30	F	31	30	Naïve	3,0	
**BL65**	23	F	25	24	Naïve	2,0	
**BL66**	44	F	45	37	Switcher	2,0	
**BL67**	25	F	26	25	Naïve	1,0	
**BL50**	20	M	22	20	Switcher	2,0	
**BL51**	38	F	39	24	Switcher	3,0	
**BL52**	46	M	47	34	Switcher	3,0	
**BL53**	39	F	40	33	Switcher	3,0	
**BL54**	32	F	34	32	Switcher	2,0	
**BL55**	32	M	33	30	Switcher	2,0	
**BL56**	36	F	37	33	Switcher	2,0	
**BL57**	61	F	63	47	Switcher	2,0	
**BL58**	40	M	42	41	Switcher	2,0	
**BL59**	41	F	42	36	Switcher	3,0	
**BL60**	27	F	28	20	Switcher	3,0	
**BL61**	44	F	45	38	Switcher	3,5	
**BL62**	40	F	41	41	Naïve	3,0	
**BL68**	36	F	37	33	Switcher	3,0	

Supplementary Table S1b. Demographic characteristics of HC subjects included in the proteomic analyses.

**HC code**	**Sex**	**Age**
**HC01**	M	31
**HC02**	F	29
**HC03**	F	24
**HC04**	F	23
**HC05**	F	33
**HC06**	M	29
**HC07**	F	28
**HC08**	M	31
**HC09**	F	27
**HC10**	M	28
**HC11**	M	25
**HC12**	F	48

**HC13**	F	46
**HC14**	F	58
**HC15**	F	55
**HC16**	F	42
**HC17**	F	53
**HC18**	F	54
**HC19**	M	27
**HC20**	F	28
**HC21**	F	41
**HC22**	F	41
**HC23**	F	37
**HC24**	F	31
**HC25**	F	35
**HC26**	M	69
**HC27**	F	63
**HC28**	F	31
**HC29**	F	34
**HC30**	F	31
**HC31**	M	58

Supplementary Table S1c. Comparison of MS-T0 characteristics between follow-up completers and non-completers.

Characteristic	Completers (n = 40)	Non-completers (n = 26)	p-value
Age at baseline, years, median (IQR)	44.0 (34.0–56.2)	39.0 (33.0–45.0)	0.124
Age at disease onset, years, median (IQR)	38.0 (32.8–51.0)	33.0 (30.0–37.8)	0.030
Baseline EDSS, median (IQR)	2.0 (1.9–3.0)	3.0 (2.0–3.0)	0.206
Female, n (%)	29 (72.5)	17 (65.4)	0.591
Male, n (%)	11 (27.5)	9 (34.6)	
Treatment-naïve, n (%)	12 (30.0)	6 (23.1)	0.585
Switcher, n (%)	28 (70.0)	20 (76.9)	
Planned DMT, n (%)			
Cladribine	5 (12.5)	7 (26.9)	
Dimethyl fumarate	13 (32.5)	5 (19.2)	
Natalizumab	3 (7.5)	1 (3.8)	
Ocrelizumab	6 (15.0)	6 (23.1)	
Ozanimod	2 (5.0)	0 (0.0)	
Teriflunomide	11 (27.5)	7 (26.9)	

Data are reported as median (interquartile range) or number (percentage), as appropriate. Completers were participants who provided both MS-T0 and MS-T1 saliva samples; non-completers provided only the MS-T0 sample. The p-value for disease-modifying therapy refers to the overall comparison of treatment distributions. EDSS, Expanded Disability Status Scale; DMT, disease-modifying therapy; IQR, interquartile range.

Supplementary Table S1d. Sensitivity analysis comparing treatment-naïve and previously treated (switcher) patients at MS-T0.

Panel A. Baseline clinical and demographic characteristics

Characteristic	Treatment-naïve (n = 18)	Switcher (n = 48)	p-value
Age at baseline, years	40.0 (31.5–56.5)	42.0 (35.5–47.2)	0.7187
Age at onset, years	39.5 (30.5–55.5)	35.5 (31.5–40.2)	0.3950
Disease duration, years	1.0 (1.0–1.0)	4.0 (2.0–7.0)	<0.0001
EDSS at baseline	2.2 (2.0–3.0)	2.0 (2.0–3.0)	0.4991
Sex, female	14/18 (77.8%)	32/48 (66.7%)	0.5494

Data are median (interquartile range) or number/total (percentage), as appropriate. Continuous variables were compared using two-sided Mann-Whitney U tests and sex using Fisher's exact test. Disease duration was calculated as age at baseline minus age at disease onset. EDSS, Expanded Disability Status Scale.

Panel B. Summary of the proteomic sensitivity analysis

Analysis item	Result
Proteins tested	623
Proteins with nominal p < 0.05	54
Proteins with Benjamini-Hochberg FDR < 0.05	0
Minimum nominal p-value	0.0045
Minimum FDR-adjusted p-value	0.4562

For each of the 623 proteins, MS-T0 abundance was compared between treatment-naïve and switcher patients using a Wilcoxon rank-sum test. Benjamini-Hochberg correction was applied across the 623 protein-wise comparisons. No protein remained significant after FDR correction. FDR, false discovery rate; HC, healthy controls; MS-T0, patients with relapsing-remitting multiple sclerosis at baseline.

Supplementary Table S2a. Wilcoxon rank-sum test comparisons between the three groups: MS-T0, MS-T1, and HC. Paired comparions between MS-T0 and MS-T1 by the Wilcoxon signed rank test are also shown (last column). For each comparison the significant p-value with a 1% FDR (cut-off 0.00165) and the direction of the change are reported. Zero values indicate p-values < 0.0001.

	**MS-T0 vs HC**		**MS-T1 vs HC**		**MS-T0 vs MS-T1**		
**UniProt code**	change	p	change	p	change	unpaired p	paired p
P09622	MS-T0>HC	0.0006	MS-T1>HC	0.0000	MS-T0<MS-T1	0.0013	0.0010
P60900	MS-T0>HC	0.0000	MS-T1>HC	0.0000			
P67936	MS-T0>HC	0.0001	MS-T1>HC	0.0000			
O75368	MS-T0>HC	0.0001	MS-T1>HC	0.0000			
Q15843	MS-T0>HC	0.0002	MS-T1>HC	0.0000			
P23141	MS-T0>HC	0.0001	MS-T1>HC	0.0000			
P17174	MS-T0>HC	0.0001	MS-T1>HC	0.0001			
P09211	MS-T0>HC	0.0000	MS-T1>HC	0.0001			
P10599	MS-T0>HC	0.0000	MS-T1>HC	0.0001			
P60174	MS-T0>HC	0.0003	MS-T1>HC	0.0001			
P61604	MS-T0>HC	0.0000	MS-T1>HC	0.0001			
P30086	MS-T0>HC	0.0001	MS-T1>HC	0.0005			
P07355	MS-T0>HC	0.0005	MS-T1>HC	0.0006			
O60218	MS-T0>HC	0.0000	MS-T1>HC	0.0007			
P06396	MS-T0>HC	0.0007	MS-T1>HC	0.0011			
P04075	MS-T0>HC	0.0000	MS-T1>HC	0.0013			
P20810	MS-T0>HC	0.0007	MS-T1>HC	0.0015			
Q01813	MS-T0<HC	0.0000	MS-T1<HC	0.0000			
Q96BQ1	MS-T0<HC	0.0006	MS-T1<HC	0.0000			
P49642	MS-T0<HC	0.0000	MS-T1<HC	0.0000			
P22079	MS-T0<HC	0.0001	MS-T1<HC	0.0000			
P01619	MS-T0<HC	0.0007	MS-T1<HC	0.0000			
P19021	MS-T0<HC	0.0002	MS-T1<HC	0.0000			
Q9NXH9	MS-T0<HC	0.0000	MS-T1<HC	0.0000			
P00492	MS-T0<HC	0.0004	MS-T1<HC	0.0000			
Q14204	MS-T0<HC	0.0013	MS-T1<HC	0.0000			
O95202	MS-T0<HC	0.0000	MS-T1<HC	0.0006			
P00338	MS-T0<HC	0.0001	MS-T1<HC	0.0010			
O60437	MS-T0<HC	0.0000	MS-T1<HC	0.0015			
P28838	MS-T0>HC	0.0000			MS-T0>MS-T1	0.0000	0.0000
P05109	MS-T0>HC	0.0000			MS-T0>MS-T1	0.0000	0.0000
P62258	MS-T0>HC	0.0000			MS-T0>MS-T1	0.0000	0.0000
P06703	MS-T0>HC	0.0001			MS-T0>MS-T1	0.0000	0.0000
P13987	MS-T0>HC	0.0001			MS-T0>MS-T1	0.0000	0.0000
P37802	MS-T0>HC	0.0000			MS-T0>MS-T1	0.0000	0.0000
Q9UJ70	MS-T0>HC	0.0001			MS-T0>MS-T1	0.0000	0.0000
Q08188	MS-T0>HC	0.0000			MS-T0>MS-T1	0.0000	0.0000

	MS-T0 vs HC		MS-T1 vs HC		MS-T0 vs MS-T1		
**UniProt code**	change	p	change	p	change	unpaired p	paired p
P30740	MS-T0>HC	0.0000			MS-T0>MS-T1	0.0000	0.0000
P50395	MS-T0>HC	0.0003			MS-T0>MS-T1	0.0000	0.0000
P27482	MS-T0>HC	0.0000			MS-T0>MS-T1	0.0000	0.0001
P13798	MS-T0>HC	0.0000			MS-T0>MS-T1	0.0000	0.0000
P20160	MS-T0>HC	0.0000			MS-T0>MS-T1	0.0000	0.0000
P13796	MS-T0>HC	0.0000			MS-T0>MS-T1	0.0000	0.0002
O43707	MS-T0>HC	0.0000			MS-T0>MS-T1	0.0000	0.0001
P02545	MS-T0>HC	0.0001			MS-T0>MS-T1	0.0001	0.0010
P62937	MS-T0>HC	0.0013			MS-T0>MS-T1	0.0001	0.0003
Q01469	MS-T0>HC	0.0000			MS-T0>MS-T1	0.0002	
Q9NYL9	MS-T0>HC	0.0011			MS-T0>MS-T1	0.0002	0.0000
P62805	MS-T0>HC	0.0001			MS-T0>MS-T1	0.0003	0.0000
O75367	MS-T0>HC	0.0002			MS-T0>MS-T1	0.0004	0.0000
P16401	MS-T0>HC	0.0001			MS-T0>MS-T1	0.0004	
O15145	MS-T0>HC	0.0007			MS-T0>MS-T1	0.0005	
P09429	MS-T0>HC	0.0000			MS-T0>MS-T1	0.0006	
P20618	MS-T0>HC	0.0001			MS-T0>MS-T1	0.0006	
Q05682	MS-T0>HC	0.0001			MS-T0>MS-T1	0.0015	
Q02818	MS-T0<HC	0.0001			MS-T0<MS-T1	0.0000	0.0000
P00751	MS-T0<HC	0.0003			MS-T0<MS-T1	0.0000	0.0000
P02750	MS-T0<HC	0.0000			MS-T0<MS-T1	0.0000	0.0000
A0A0B4J1X5	MS-T0<HC	0.0000			MS-T0<MS-T1	0.0000	0.0000
Q00610	MS-T0<HC	0.0001			MS-T0<MS-T1	0.0000	0.0000
P09960	MS-T0<HC	0.0000			MS-T0<MS-T1	0.0000	0.0002
A0A0C4DH31	MS-T0<HC	0.0000			MS-T0<MS-T1	0.0000	0.0001
P12830	MS-T0<HC	0.0001			MS-T0<MS-T1	0.0000	0.0012
P15586	MS-T0<HC	0.0000			MS-T0<MS-T1	0.0001	
Q16555	MS-T0<HC	0.0008			MS-T0<MS-T1	0.0001	0.0010
P21579	MS-T0<HC	0.0000			MS-T0<MS-T1	0.0009	
P05091	MS-T0<HC	0.0000			MS-T0<MS-T1	0.0013	0.0005
Q09666	MS-T0>HC	0.0000					
P04080	MS-T0>HC	0.0000					
Q14CN2	MS-T0>HC	0.0000					
P37837	MS-T0>HC	0.0000					
O00748	MS-T0>HC	0.0000					
Q06830	MS-T0>HC	0.0000					
P0DP25	MS-T0>HC	0.0000					
Q6ZVX7	MS-T0>HC	0.0000					
P14174	MS-T0>HC	0.0001					
P27797	MS-T0>HC	0.0001					
Q96HE7	MS-T0>HC	0.0001					
P30838	MS-T0>HC	0.0001					
P06733	MS-T0>HC	0.0001					
P07108	MS-T0>HC	0.0002					
P02768	MS-T0>HC	0.0002					

	MS-T0 vs HC		MS-T1 vs HC		MS-T0 vs MS-T1		
**UniProt code**	change	p	change	p	change	unpaired p	paired p
P32119	MS-T0>HC	0.0002					
P05164	MS-T0>HC	0.0002					0.0000
P30101	MS-T0>HC	0.0002					
P29508	MS-T0>HC	0.0002					
Q6UWP8	MS-T0>HC	0.0002					
A8K2U0	MS-T0>HC	0.0003					
P41218	MS-T0>HC	0.0004					
P11021	MS-T0>HC	0.0004					
P80188	MS-T0>HC	0.0005					
P52209	MS-T0>HC	0.0006					
Q92817	MS-T0>HC	0.0010					
Q9BRA2	MS-T0>HC	0.0010					
P02788	MS-T0>HC	0.0011					0.0009
P26583	MS-T0>HC	0.0011					0.0007
O95274	MS-T0>HC	0.0013					
P04083	MS-T0<HC	0.0000					
P34096	MS-T0<HC	0.0000					
P62993	MS-T0<HC	0.0000					
P35659	MS-T0<HC	0.0000					
Q9Y265	MS-T0<HC	0.0000					
P13688	MS-T0<HC	0.0001					
P62879	MS-T0<HC	0.0001					
P39656	MS-T0<HC	0.0002					
P01034	MS-T0<HC	0.0002					
P02814	MS-T0<HC	0.0002					
Q08380	MS-T0<HC	0.0003					
P0DOX4	MS-T0<HC	0.0004					
P80303	MS-T0<HC	0.0005					
P02790	MS-T0<HC	0.0005					
P59998	MS-T0<HC	0.0007					
Q13813	MS-T0<HC	0.0007					
Q9Y2J8	MS-T0<HC	0.0007					
Q96DR8	MS-T0<HC	0.0008					
P14868	MS-T0<HC	0.0008					
O75347			MS-T1>HC	0.0002	MS-T0<MS-T1	0.0000	0.0000
Q13510			MS-T1>HC	0.0000	MS-T0<MS-T1	0.0000	0.0001
Q15084			MS-T1>HC	0.0000	MS-T0<MS-T1	0.0000	0.0002
P00441			MS-T1>HC	0.0003	MS-T0<MS-T1	0.0000	0.0000
Q13126			MS-T1>HC	0.0006	MS-T0<MS-T1	0.0000	0.0012
P06744			MS-T1>HC	0.0000	MS-T0<MS-T1	0.0003	0.0004
Q9H299			MS-T1>HC	0.0000	MS-T0<MS-T1	0.0009	
P11940			MS-T1<HC	0.0001	MS-T0>MS-T1	0.0000	0.0000
P78417			MS-T1<HC	0.0006	MS-T0>MS-T1	0.0000	0.0000
P23284			MS-T1<HC	0.0015	MS-T0>MS-T1	0.0000	0.0000
Q9HD89			MS-T1<HC	0.0000	MS-T0>MS-T1	0.0000	0.0001

	MS-T0 vs HC		MS-T1 vs HC		MS-T0 vs MS-T1		
**UniProt code**	change	p	change	p	change	unpaired p	paired p
P36871			MS-T1<HC	0.0013	MS-T0>MS-T1	0.0000	0.0007
P36222			MS-T1<HC	0.0013	MS-T0>MS-T1	0.0000	0.0001
O95336			MS-T1>HC	0.0000			
P40926			MS-T1>HC	0.0000			0.0013
P49862			MS-T1>HC	0.0004			
P13639			MS-T1>HC	0.0016			
P49411			MS-T1<HC	0.0000			
P35998			MS-T1<HC	0.0000			
O60488			MS-T1<HC	0.0001			
P55058			MS-T1<HC	0.0003			0.0004
P50990			MS-T1<HC	0.0007			
Q9UQ80			MS-T1<HC	0.0010			
P63027			MS-T1<HC	0.0011			
Q8NBJ4			MS-T1<HC	0.0015			
P31946					MS-T0<MS-T1	0.0000	0.0001
Q15847					MS-T0<MS-T1	0.0000	0.0000
P14618					MS-T0<MS-T1	0.0000	0.0001
P07910					MS-T0<MS-T1	0.0000	
Q9UJU6					MS-T0<MS-T1	0.0000	
P30085					MS-T0<MS-T1	0.0000	0.0001
P38606					MS-T0<MS-T1	0.0000	0.0004
Q86U42					MS-T0<MS-T1	0.0000	
Q9Y490					MS-T0<MS-T1	0.0000	0.0001
P23528					MS-T0<MS-T1	0.0000	0.0003
Q04917					MS-T0<MS-T1	0.0001	
Q13185					MS-T0<MS-T1	0.0001	
P18510					MS-T0<MS-T1	0.0001	0.0000
P61916					MS-T0<MS-T1	0.0001	0.0014
P78371					MS-T0<MS-T1	0.0001	0.0005
P07437					MS-T0<MS-T1	0.0001	
O14745					MS-T0<MS-T1	0.0001	
P51991					MS-T0<MS-T1	0.0001	0.0001
P49368					MS-T0<MS-T1	0.0002	0.0007
O60506					MS-T0<MS-T1	0.0002	
P04196					MS-T0<MS-T1	0.0002	
Q92499					MS-T0<MS-T1	0.0003	
P27105					MS-T0<MS-T1	0.0003	
P39687					MS-T0<MS-T1	0.0004	
P38646					MS-T0<MS-T1	0.0004	0.0009
P10809					MS-T0<MS-T1	0.0005	0.0005
P13693					MS-T0<MS-T1	0.0006	
P62495					MS-T0<MS-T1	0.0007	
P02671					MS-T0<MS-T1	0.0007	0.0000
O00764					MS-T0<MS-T1	0.0008	
Q15942					MS-T0<MS-T1	0.0008	

		**MS-T0 vs HC**			**MS-T1 vs HC**			**MS-T0 vs MS-T1**		
**UniProt code**		change	p		change	p		change	unpaired p	paired p
P06753								MS-T0<MS-T1	0.0008	
P52790								MS-T0<MS-T1	0.0008	
P02766								MS-T0<MS-T1	0.0009	0.0014
O43175								MS-T0<MS-T1	0.0009	
P07339								MS-T0<MS-T1	0.0011	
P07195								MS-T0<MS-T1	0.0012	
P32320								MS-T0<MS-T1	0.0013	0.0006
P68366								MS-T0<MS-T1	0.0015	
Q14116								MS-T0>MS-T1	0.0000	0.0000
P07305								MS-T0>MS-T1	0.0000	0.0000
P31997								MS-T0>MS-T1	0.0000	0.0000
P07384								MS-T0>MS-T1	0.0000	0.0000
Q9UBC9								MS-T0>MS-T1	0.0000	0.0000
O15144								MS-T0>MS-T1	0.0000	0.0003
O60645								MS-T0>MS-T1	0.0000	0.0013
Q99536								MS-T0>MS-T1	0.0000	0.0003
P60660								MS-T0>MS-T1	0.0001	0.0004
Q9BYT8								MS-T0>MS-T1	0.0001	0.0001
A0A0J9YX35								MS-T0>MS-T1	0.0001	0.0013
P17931								MS-T0>MS-T1	0.0002	0.0011
P40121								MS-T0>MS-T1	0.0002	0.0010
P17213								MS-T0>MS-T1	0.0003	0.0009
P25311								MS-T0>MS-T1	0.0003	0.0000
P01703								MS-T0>MS-T1	0.0003	
Q9P258								MS-T0>MS-T1	0.0003	
P22735								MS-T0>MS-T1	0.0005	
P26641								MS-T0>MS-T1	0.0005	
P02787								MS-T0>MS-T1	0.0007	0.0001
P58062								MS-T0>MS-T1	0.0008	
P0DMV9								MS-T0>MS-T1	0.0009	0.0002
Q08945								MS-T0>MS-T1	0.0010	
P11215								MS-T0>MS-T1	0.0010	0.0002
Q6P5S2								MS-T0>MS-T1	0.0010	
O95171								MS-T0>MS-T1	0.0011	0.0004
P47756								MS-T0>MS-T1	0.0013	
Q96DA0								MS-T0>MS-T1	0.0015	
O60235								MS-T0>MS-T1	0.0015	0.0007
P05107								MS-T0>MS-T1		0.0001
P10153								MS-T0>MS-T1		0.0009
P12277								MS-T0<MS-T1		0.0003
P15153								MS-T0>MS-T1		0.0014
P15515								MS-T0<MS-T1		0.0012
P18669								MS-T0>MS-T1		0.0015
P36952								MS-T0>MS-T1		0.0010
P49721								MS-T0<MS-T1		0.0002

		MS-T0 vs HC			MS-T1 vs HC			MS-T0 vs MS-T1		
**UniProt code**		change	p		change	p		change	unpaired p	paired p
P59666								MS-T0>MS-T1		0.0002
P60842								MS-T0<MS-T1		0.0008
P61586								MS-T0>MS-T1		0.0003
P61626								MS-T0>MS-T1		0.0005
Q14019								MS-T0>MS-T1		0.0001
Q14508								MS-T0<MS-T1		0.0011
Q8N6Q3								MS-T0>MS-T1		0.0008

Supplementary Table S2b. Spearman rank correlations were calculated between each clinical or demographic variable and the abundance of each of the 623 proteins, separately at baseline (MS-T0), six months after disease-modifying therapy exposure (MS-T1), and for the paired abundance changes between the two time points. Benjamini–Hochberg correction was applied separately within each combination. The last two columns report the number of proteins with a nominal p-value < 0.05 and the number remaining significant at a false discovery rate < 0.05, respectively.

Proteomic measure	Clinical variable	Patients, n	Proteins tested	Proteins with nominal p < 0.05, n	Proteins with BH-FDR < 0.05, n
MS-T0 abundance	Year of disease onset	66	623	68	0
MS-T0 abundance	Age at disease onset	66	623	82	8
MS-T0 abundance	Age at baseline	66	623	77	5
MS-T0 abundance	Disease duration at baseline	66	623	69	0
MS-T0 abundance	Baseline EDSS	66	623	13	0
MS-T1 abundance	Year of disease onset	40	623	40	0
MS-T1 abundance	Age at disease onset	40	623	62	0
MS-T1 abundance	Age at baseline	40	623	43	0
MS-T1 abundance	Disease duration at baseline	40	623	30	0
MS-T1 abundance	Baseline EDSS	40	623	29	0
Paired change (MS-T1 minus MS-T0)	Year of disease onset	40	623	29	0
Paired change (MS-T1 minus MS-T0)	Age at disease onset	40	623	41	0

Paired change (MS-T1 minus MS-T0)	Age at baseline	40	623	32	0
Paired change (MS-T1 minus MS-T0)	Disease duration at baseline	39	623	37	0
Paired change (MS-T1 minus MS-T0)	Baseline EDSS	40	623	63	0

EDSS, Expanded Disability Status Scale; FDR, false discovery rate.

Supplementary Table S2c. FDR-significant associations between salivary protein abundance at MS-T0 and age-related clinical variables.

Clinical variable	Protein name (UniProt code)	n	Spearman rho	Nominal p-value	FDR-adjusted p-value
Age at baseline	Cysteine-rich secretory protein 3 (P54108)	66	-0.463	0.000091	0.0388
Age at baseline	Progranulin (P28799)	66	-0.455	0.000124	0.0388
Age at baseline	Protein LEG1 homolog (Q6P5S2)	66	-0.438	0.000237	0.0475
Age at baseline	SPARC-like protein 1 (Q14515)	66	-0.425	0.000380	0.0475
Age at baseline	Mucin-7 (Q8TAX7)	66	-0.425	0.000381	0.0475
Age at disease onset	Myosin-9 (P35579)	65	0.443	0.000220	0.0400
Age at disease onset	Myeloperoxidase (P05164)	65	0.436	0.000280	0.0400
Age at disease onset	Lactotransferrin (P02788)	65	0.434	0.000306	0.0400
Age at disease onset	Protein LEG1 homolog (Q6P5S2)	65	-0.430	0.000354	0.0400
Age at disease onset	Core histone macro-H2A.1 (O75367)	65	0.429	0.000366	0.0400
Age at disease onset	Ras GTPase-activating-like protein IQGAP1 (P46940)	65	0.427	0.000395	0.0400
Age at disease onset	Moesin (P26038)	65	0.423	0.000450	0.0400
Age at disease onset	Cysteine-rich secretory protein 3 (P54108)	65	-0.413	0.000630	0.0491

Supplementary Table S3. Relative importance of proteins for Random Forest classification. The table reports the Mean Decrease Accuracy (MDA) of the 50 proteins selected by the Boruta algorithm for RF. The MDA reflects the reduction in classification accuracy observed when the values of a given protein are randomly permuted; therefore, higher values indicate a stronger contribution of that protein to the classification. The MDA index is calculated for the overall classification model (global, in decreasing order) and separately for each class: HC, MS-T0, and MS-T1.

	MDA MeanDecreaseAccuracy			
	global	HC	MS-T0	MS-T1
Q01813	0,0404	0,0915	0,0006	0,1001
P28838	0,0301	0,0157	0,0323	0,0343
P62258	0,0227	0,0577	0,0118	0,0011
P11940	0,0221	0,0038	0,0160	0,0467
P60900	0,0163	0,0031	0,0205	0,0400
A0A0B4J1X5	0,0142	0,0219	0,0146	0,0082
P37802	0,0119	0,0045	0,0035	0,0304
P13798	0,0109	0,0133	0,0104	0,0144
Q13510	0,0105	0,0166	0,0126	0,0042
P05109	0,0078	0,0189	0,0050	0,0059
P06703	0,0078	0,0167	0,0069	0,0024
Q14116	0,0075	0,0054	0,0064	0,0127
P19021	0,0074	0,0070	0,0083	0,0076
P27482	0,0072	0,0156	0,0056	0,0011
O75347	0,0071	0,0021	0,0039	0,0151
P13987	0,0068	0,0003	0,0058	0,0146
Q9UJ70	0,0065	0,0007	0,0077	0,0111
P02750	0,0063	0,0095	-0,0002	0,0096
Q9NXH9	0,0060	0,0111	0,0047	0,0031
P00751	0,0060	0,0026	0,0076	0,0076
P07384	0,0056	-0,0035	0,0020	0,0189
P30740	0,0053	0,0014	0,0070	0,0079
Q02818	0,0053	0,0091	0,0047	0,0023
P21579	0,0049	0,0095	0,0055	0,0005
O43707	0,0048	0,0092	0,0028	0,0035
P31946	0,0048	0,0069	0,0010	0,0066
P39656	0,0043	0,0053	0,0035	0,0057
Q15084	0,0043	0,0003	0,0073	0,0066
P78417	0,0042	0,0098	0,0006	0,0038

P10809	0,0041	0,0000	0,0052	0,0088
P42785	0,0040	0,0044	0,0005	0,0090
P09622	0,0039	0,0083	0,0002	0,0047
Q08188	0,0037	0,0096	0,0013	0,0022
P23284	0,0035	0,0000	0,0029	0,0101
O95336	0,0034	0,0034	0,0043	0,0041
P06744	0,0033	0,0066	0,0025	0,0024
P12830	0,0032	0,0006	0,0058	0,0050
P05091	0,0031	0,0036	0,0000	0,0078
P13796	0,0030	0,0000	0,0046	0,0067
Q15847	0,0029	0,0004	0,0031	0,0063
Q15843	0,0029	0,0002	0,0046	0,0041
P49642	0,0029	0,0008	0,0014	0,0065
O75368	0,0027	0,0000	0,0029	0,0056
P62993	0,0027	0,0026	0,0033	0,0024
P02545	0,0026	0,0018	0,0024	0,0036
P36871	0,0026	0,0034	0,0036	0,0001
P15586	0,0026	0,0016	0,0049	0,0004
P49411	0,0023	0,0037	0,0012	0,0014
P02790	0,0019	0,0018	0,0039	0,0000
Q9Y6W5	0,0017	0,0015	0,0023	0,0010

Supplemental Figure S1: Illustrative classification tree generated within the RF outputs. For visualization purposes, the tree was constructed using proteins emerged as the most important in the classification according to Mean Decrease Accuracy (MDA); Internal nodes show UniProt accession codes and split points for the processed protein abundance values; the corresponding protein names are reported in Supplementary Table S3. Terminal nodes indicate the predicted classes. “Y” indicates that the condition specified at the node is true, whereas “N” indicates that it is not true. In the standard RF procedure, each tree is randomly generated using only a limited number of variables and two-thirds of subjects. Samples not included contribute to estimation of the out-of-bag error. Some proteins may therefore appear at different split points within a tree, while other trees (compressively 1500) may use different proteins in the split points. This explains the conditional, nonlinear and multivariate nature of RF classification and helps explain why the results of the RF occasionally diverge from those of univariate tests. HC, healthy controls; MS-T0, patients with relapsing-remitting multiple sclerosis at baseline; MS-T1, patients after six months of disease-modifying therapy.

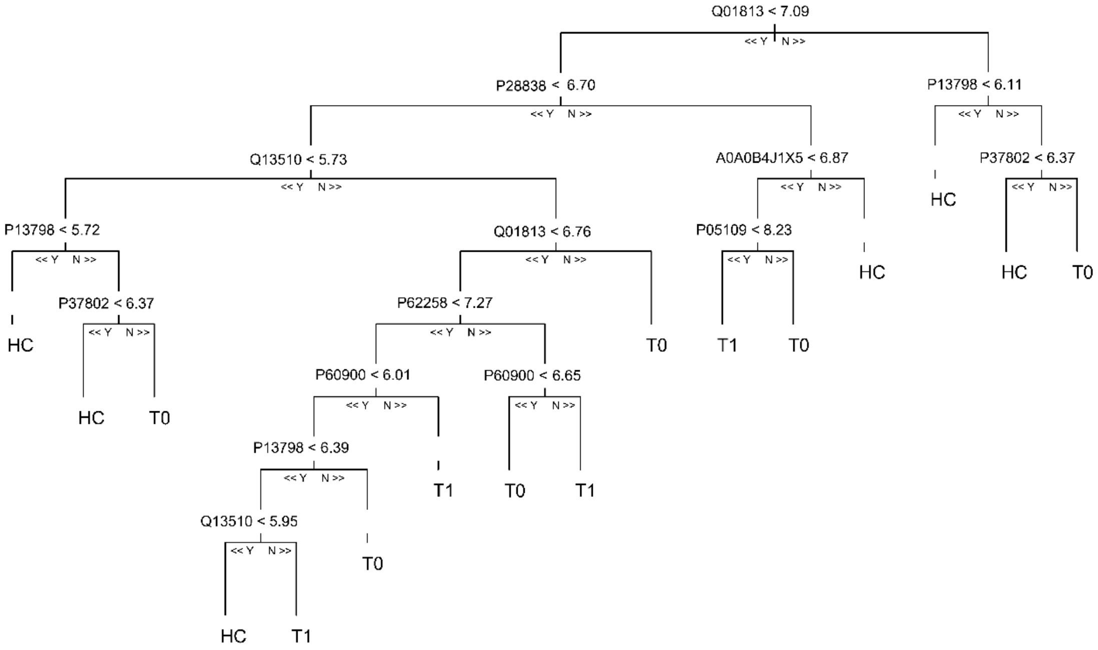

Supplemental Figure S2: Heatmap of the 209 proteins with significant abundance differences among HC, MS-T0 and MS-T1. Protein abundances were row-wise standardized (mean = 0, SD = 1) and clustered using hierarchical clustering, to obtain homogeneous subsets. Colors represent relative protein abundance, ranging from low (green) to high abundance (red).

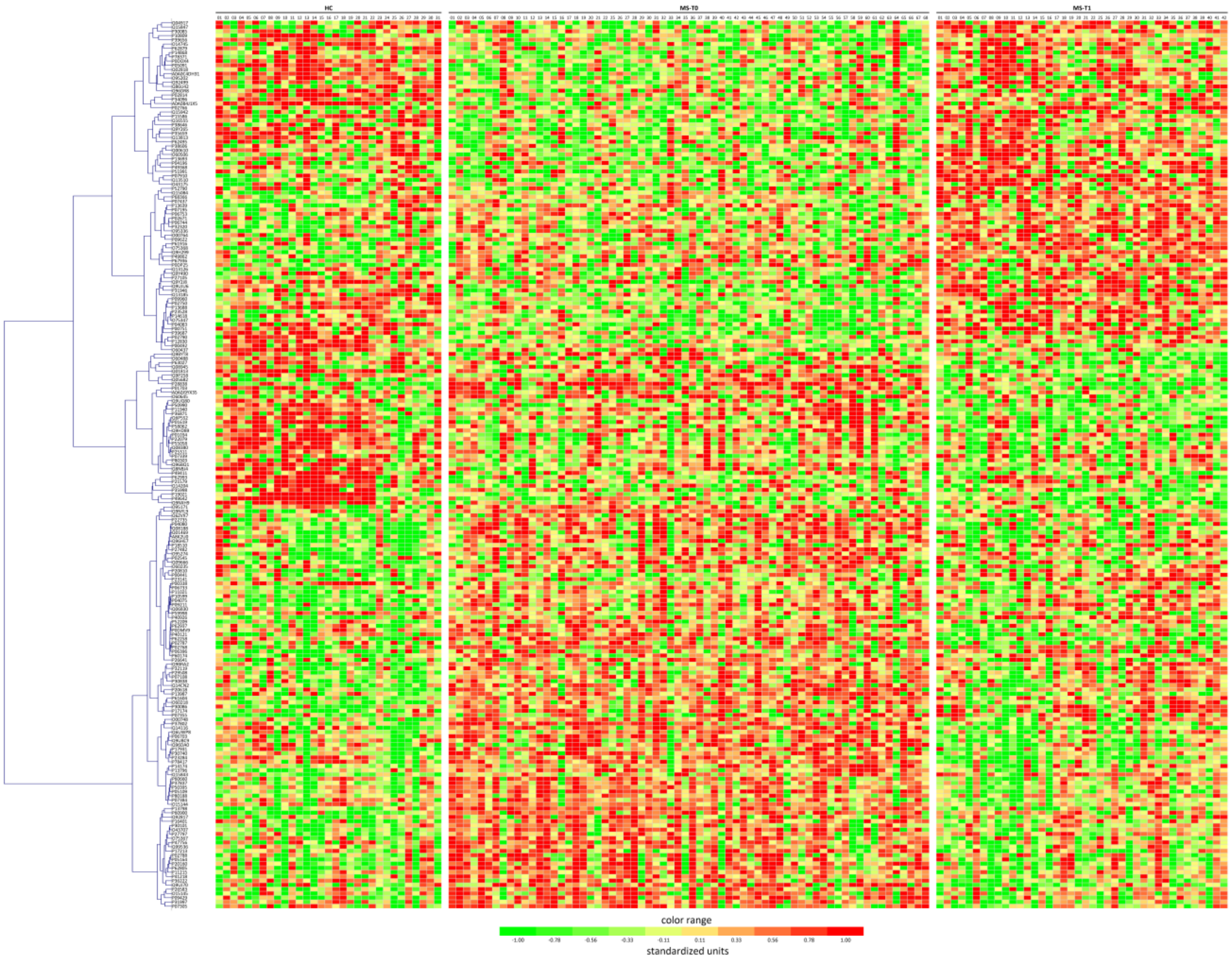

Supplementary Table S4. Protein composition and Reactome enrichment of proteomic patterns. Protein names are reported together with UniProt accession codes.

Table S4a. Persistent RRMS signature: protein composition

Directional subset	Direction / selection criterion	UniProt code	Protein name
Persistently increased	MS-T0 > HC; MS-T1 > HC; MS-T0 ≈ MS-T1	P60900	Proteasome subunit alpha type-6
Persistently increased	MS-T0 > HC; MS-T1 > HC; MS-T0 ≈ MS-T1	P67936	Tropomyosin alpha-4 chain
Persistently increased	MS-T0 > HC; MS-T1 > HC; MS-T0 ≈ MS-T1	O75368	SH3 domain-binding glutamic acid-rich-like protein 3
Persistently increased	MS-T0 > HC; MS-T1 > HC; MS-T0 ≈ MS-T1	Q15843	NEDD8
Persistently increased	MS-T0 > HC; MS-T1 > HC; MS-T0 ≈ MS-T1	P23141	Liver carboxylesterase 1
Persistently increased	MS-T0 > HC; MS-T1 > HC; MS-T0 ≈ MS-T1	P17174	Aspartate aminotransferase, cytoplasmic
Persistently increased	MS-T0 > HC; MS-T1 > HC; MS-T0 ≈ MS-T1	P09211	Glutathione S-transferase P
Persistently increased	MS-T0 > HC; MS-T1 > HC; MS-T0 ≈ MS-T1	P10599	Thioredoxin
Persistently increased	MS-T0 > HC; MS-T1 > HC; MS-T0 ≈ MS-T1	P60174	Triosephosphate isomerase
Persistently increased	MS-T0 > HC; MS-T1 > HC; MS-T0 ≈ MS-T1	P61604	10 kDa heat shock protein, mitochondrial
Persistently increased	MS-T0 > HC; MS-T1 > HC; MS-T0 ≈ MS-T1	P30086	Phosphatidylethanolamine-binding protein 1
Persistently increased	MS-T0 > HC; MS-T1 > HC; MS-T0 ≈ MS-T1	P07355	Annexin A2
Persistently increased	MS-T0 > HC; MS-T1 > HC; MS-T0 ≈ MS-T1	O60218	Aldo-keto reductase family 1 member B10
Persistently increased	MS-T0 > HC; MS-T1 > HC; MS-T0 ≈ MS-T1	P06396	Gelsolin
Persistently increased	MS-T0 > HC; MS-T1 > HC; MS-T0 ≈ MS-T1	P04075	Fructose-bisphosphate aldolase A
Persistently increased	MS-T0 > HC; MS-T1 > HC; MS-T0 ≈ MS-T1	P20810	Calpastatin
Persistently decreased	MS-T0 < HC; MS-T1 < HC; MS-T0 ≈ MS-T1	Q01813	ATP-dependent 6-phosphofructokinase, platelet type
Persistently decreased	MS-T0 < HC; MS-T1 < HC; MS-T0 ≈ MS-T1	Q96BQ1	Protein FAM3D
Persistently decreased	MS-T0 < HC; MS-T1 < HC; MS-T0 ≈ MS-T1	P49642	DNA primase large subunit
Persistently decreased	MS-T0 < HC; MS-T1 < HC; MS-T0 ≈ MS-T1	P22079	Lactoperoxidase
Persistently decreased	MS-T0 < HC; MS-T1 < HC; MS-T0 ≈ MS-T1	P01619	Immunoglobulin kappa variable 3-20
Persistently decreased	MS-T0 < HC; MS-T1 < HC; MS-T0 ≈ MS-T1	P19021	Peptidyl-glycine alpha-amidating monooxygenase
Persistently decreased	MS-T0 < HC; MS-T1 < HC; MS-T0 ≈ MS-T1	Q9NXH9	tRNA 2,2-dimethylguanosine-26 methyltransferase
Persistently decreased	MS-T0 < HC; MS-T1 < HC; MS-T0 ≈ MS-T1	P00492	Hypoxanthine-guanine phosphoribosyltransferase
Persistently decreased	MS-T0 < HC; MS-T1 < HC; MS-T0 ≈ MS-T1	Q14204	Cytoplasmic dynein 1 heavy chain 1
Persistently decreased	MS-T0 < HC; MS-T1 < HC; MS-T0 ≈ MS-T1	O95202	LETM1 and EF-hand domain-containing protein 1, mitochondrial
Persistently decreased	MS-T0 < HC; MS-T1 < HC; MS-T0 ≈ MS-T1	P00338	L-lactate dehydrogenase A chain
Persistently decreased	MS-T0 < HC; MS-T1 < HC; MS-T0 ≈ MS-T1	O60437	Peptidyl-tRNA hydrolase 2, mitochondrial

≈ symbol conventionally means the lack of statistically significant difference.

Table S4b. Baseline-attenuation signature: significant Reactome pathways and contributing proteins

Reactome pathway / ID	FDR-adjusted p-value	Intersection size	Proteins decreased in MS-T1	Proteins increased in MS-T1	Interpretation note
Neutrophil degranulation REAC:R-HSA-6798695	7.15e-08	13	P05109 — Protein S100-A8; P13987 — CD59 glycoprotein; P30740 — Leukocyte elastase inhibitor; P50395 — Rab GDP dissociation inhibitor beta; P13798 — Acylamino-acid-releasing enzyme; P20160 — Azurocidin; P62937 — Peptidyl-prolyl cis-trans isomerase A; Q01469 —	P02750 — Leucine-rich alpha-2-glycoprotein; P09960 — Leukotriene A-4 hydrolase; P15586 — N-acetylglucosamine-6-sulfatase	Mainly supported by proteins decreased in MS-T1.

			Fatty acid-binding protein 5; P09429 — High mobility group protein B1; P20618 — Proteasome subunit beta type-1		
Innate Immune System REAC:R-HSA-168249	1.33e-05	15	P05109 — Protein S100-A8; P13987 — CD59 glycoprotein; P30740 — Leukocyte elastase inhibitor; P50395 — Rab GDP dissociation inhibitor beta; P13798 — Acylamino-acid-releasing enzyme; P20160 — Azurocidin; P62937 — Peptidyl-prolyl cis-trans isomerase A; Q01469 — Fatty acid-binding protein 5; O15145 — Actin-related protein 2/3 complex subunit 3; P09429 — High mobility group protein B1; P20618 — Proteasome subunit beta type-1	P00751 — Complement factor B; P02750 — Leucine-rich alpha-2-glycoprotein; P09960 — Leukotriene A-4 hydrolase; P15586 — N-acetylglucosamine-6-sulfatase	Predominantly supported by proteins decreased in MS-T1.
Immune System REAC:R-HSA-168256	2.25e-04	18	P05109 — Protein S100-A8; P13987 — CD59 glycoprotein; P30740 — Leukocyte elastase inhibitor; P50395 — Rab GDP dissociation inhibitor beta; P13798 — Acylamino-acid-releasing enzyme; P20160 — Azurocidin; P13796 — Plastin-2; P62937 — Peptidyl-prolyl cis-trans isomerase A; Q01469 — Fatty acid-binding protein 5; O15145 — Actin-related protein 2/3 complex subunit 3; P09429 — High mobility group protein B1; P20618 — Proteasome subunit beta type-1	P00751 — Complement factor B; P02750 — Leucine-rich alpha-2-glycoprotein; Q00610 — Clathrin heavy chain 1; P09960 — Leukotriene A-4 hydrolase; P12830 — Cadherin-1; P15586 — N-acetylglucosamine-6-sulfatase	Mixed contribution, with prevalence of proteins decreased in MS-T1.
Apoptosis REAC:R-HSA-109581	1.39e-02	5	P62258 — 14-3-3 protein epsilon; P16401 — Histone H1.5; P09429 — High mobility group protein B1; P20618 — Proteasome subunit beta type-1	P12830 — Cadherin-1	Mainly supported by proteins decreased in MS-T1.
Programmed Cell Death REAC:R-HSA-5357801	2.43e-02	5	P62258 — 14-3-3 protein epsilon; P16401 — Histone H1.5; P09429 — High mobility group protein B1; P20618 — Proteasome subunit beta type-1	P12830 — Cadherin-1	Mainly supported by proteins decreased in MS-T1.
Apoptotic execution phase REAC:R-HSA-75153	3.00e-02	3	P16401 — Histone H1.5; P09429 — High mobility group protein B1	P12830 — Cadherin-1	Mixed contribution.
Apoptosis induced DNA fragmentation REAC:R-HSA-140342	3.95e-02	2	P16401 — Histone H1.5; P09429 — High mobility group protein B1	—	Supported only by proteins decreased in MS-T1.

Table S4c. Treatment-associated signature: significant Reactome pathways and contributing proteins

Reactome pathway / ID	FDR-adjusted p-value	Intersection size	MS-T1 increased proteins	MS-T1 decreased proteins	Interpretation note
Neutrophil degranulation REAC:R-HSA-6798695	6.61e-05	9	Q13510 — Acid ceramidase; P06744 — Glucose-6-phosphate isomerase; P13639 — Elongation factor 2	Q9HD89 — Resistin; P36871 — Phosphoglucomutase-1; P36222 — Chitinase-3-like protein 1; P35998 — 26S proteasome regulatory subunit 7; P50990 — T-complex protein 1 subunit theta; Q9UQ80 — Proliferation-associated protein 2G4	Mainly supported by MS-T1-decreased proteins.
Gene and protein expression by JAK-STAT signaling after Interleukin-12 stimulation REAC:R-HSA-8950505	6.24e-03	3	P00441 — Superoxide dismutase [Cu-Zn]; Q13126 — S-methyl-5-thioadenosine phosphorylase	P78417 — Glutathione S-transferase omega-1	Small mixed pathway, with prevalence of MS-T1-increased proteins.
Immune System REAC:R-HSA-168256	6.24e-03	13	Q13510 — Acid ceramidase; P00441 — Superoxide dismutase [Cu-Zn]; Q13126 — S-methyl-5-	P78417 — Glutathione S-transferase omega-1; Q9HD89 — Resistin;	Mixed contribution, with prevalence of MS-T1-decreased proteins.

			thioadenosine phosphorylase; P06744 — Glucose-6-phosphate isomerase; P13639 — Elongation factor 2	P36871 — Phosphoglucomutase-1; P36222 — Chitinase-3-like protein 1; P35998 — 26S proteasome regulatory subunit 7; P50990 — T-complex protein 1 subunit theta; Q9UQ80 — Proliferation-associated protein 2G4; P63027 — Vesicle-associated membrane protein 2	
Interleukin-12 signaling REAC:R-HSA-447115	9.44e-03	3	P00441 — Superoxide dismutase [Cu-Zn]; Q13126 — S-methyl-5-thioadenosine phosphorylase	P78417 — Glutathione S-transferase omega-1	Small mixed pathway.
Interleukin-12 family signaling REAC:R-HSA-6785807	1.11e-02	3	P00441 — Superoxide dismutase [Cu-Zn]; Q13126 — S-methyl-5-thioadenosine phosphorylase	P78417 — Glutathione S-transferase omega-1	Small mixed pathway.
Innate Immune System REAC:R-HSA-168249	1.11e-02	9	Q13510 — Acid ceramidase; P06744 — Glucose-6-phosphate isomerase; P13639 — Elongation factor 2	Q9HD89 — Resistin; P36871 — Phosphoglucomutase-1; P36222 — Chitinase-3-like protein 1; P35998 — 26S proteasome regulatory subunit 7; P50990 — T-complex protein 1 subunit theta; Q9UQ80 — Proliferation-associated protein 2G4	Mainly supported by MS-T1-decreased proteins.

Table S4d. Longitudinal-only remodeling signature: proteins increased in MS-T1

Reactome pathway / ID	FDR-adjusted p-value	Intersection size	Proteins increased in MS-T1	Interpretation note
Immune System REAC:R-HSA-168256	1.02e-03	11	P31946 — 14-3-3 protein beta/alpha; P14618 — Pyruvate kinase PKM; P38606 — V-type proton ATPase catalytic subunit A; P23528 — Cofilin-1; P18510 — Interleukin-1 receptor antagonist protein; P61916 — NPC intracellular cholesterol transporter 2; P78371 — T-complex protein 1 subunit beta; P38646 — Stress-70 protein, mitochondrial; P02671 — Fibrinogen alpha chain; P02766 — Transthyretin; P32320 — Cytidine deaminase	Longitudinal-only paired increase; not significantly different from HC at either time point.
Innate Immune System REAC:R-HSA-168249	1.97e-03	8	P14618 — Pyruvate kinase PKM; P38606 — V-type proton ATPase catalytic subunit A; P23528 — Cofilin-1; P61916 — NPC intracellular cholesterol transporter 2; P78371 — T-complex protein 1 subunit beta; P02671 — Fibrinogen alpha chain; P02766 — Transthyretin; P32320 — Cytidine deaminase	Longitudinal-only paired increase.
MAPK/RAF/RAS-related signaling Grouped Reactome terms	1.02e-03 to 3.44e-03	3	P31946 — 14-3-3 protein beta/alpha; Q9Y490 — Talin-1; P02671 — Fibrinogen alpha chain	Grouped because several Reactome terms were redundant and supported by the same protein subset.
Integrin-associated MAPK signaling Grouped Reactome terms	3.24e-03 to 3.44e-03	2	Q9Y490 — Talin-1; P02671 — Fibrinogen alpha chain	Grouped redundant integrin-MAPK terms.
Folding of actin by CCT/TriC REAC:R-HSA-390450	1.97e-03	2	P78371 — T-complex protein 1 subunit beta; P49368 — T-complex protein 1 subunit gamma	Longitudinal-only paired increase.

Table S4e. Longitudinal-only remodeling signature: proteins decreased in MS-T1

Reactome pathway / ID	FDR-adjusted p-value	Intersection size	Proteins decreased in MS-T1	Interpretation note
Neutrophil degranulation REAC:R-HSA-6798695	2.35e-04	7	P31997 — Carcinoembryonic antigen-related cell adhesion molecule 8; P07384 — Calpain-1 catalytic subunit; Q99536 — Synaptic vesicle membrane protein VAT-1 homolog; P17931 — Galectin-3; P17213 — Bactericidal permeability-increasing protein; P0DMV9 — Heat shock 70 kDa protein 1B; P11215 — Integrin alpha-M	Longitudinal-only paired decrease; not significantly different from HC at either time point.

Innate Immune System REAC:R-HSA-168249	3.24e-03	8	P31997 — Carcinoembryonic antigen-related cell adhesion molecule 8; P07384 — Calpain-1 catalytic subunit; O15144 — Actin-related protein 2/3 complex subunit 2; Q99536 — Synaptic vesicle membrane protein VAT-1 homolog; P17931 — Galectin-3; P17213 — Bactericidal permeability-increasing protein; P0DMV9 — Heat shock 70 kDa protein 1B; P11215 — Integrin alpha-M	Longitudinal-only paired decrease.

MS-T0, baseline RRMS samples before treatment initiation; MS-T1, RRMS samples after six months of DMT exposure; FDR, false discovery rate. For Baseline-attenuation signature, “decreased in MS-T1” indicates proteins increased at baseline relative to HC and reduced after treatment exposure, whereas “increased in MS-T1” indicates proteins reduced at baseline relative to HC and increased after treatment exposure. For treatment-associated proteins, MS-T1 increased/decreased indicates proteins not altered at MS-T0 versus HC but altered at MS-T1 versus HC. For longitudinal-only proteins, changes refer to paired MS-T0 versus MS-T1 comparisons without significant differences versus HC at either time point.
